# Cell‐Selective Delivery of RIBOTACs via an Anti‐EGFR Nanobody for Pancreatic Cancer Treatment

**DOI:** 10.1002/advs.76575

**Published:** 2026-07-16

**Authors:** Tianli Luo, Yijuan Wang, Dengwang Chen, Yinghan Zhang, Yi Shuai, Hualiang Yao, Tao Jiang, Hang Jiang, Ming Zhong, Xiangeng Wang, Ziwei Wang, Chenchen Xu, Ajay Goel, Hoi Tang MA, Feng Gao, Xin Wang

**Affiliations:** ^1^ Department of Surgery The Chinese University of Hong Kong Shatin Hong Kong SAR China; ^2^ Department of Pathology School of Clinical Medicine Li Ka Shing Faculty of Medicine The University of Hong Kong Hong Kong Hong Kong SAR China; ^3^ Department of Histology and Embryology Zunyi Medical University Zunyi China; ^4^ Department of Molecular Diagnostics and Experimental Therapeutics Beckman Research Institute of City of Hope Biomedical Research Center Monrovia California USA; ^5^ State Key Laboratory of Liver Research The University of Hong Kong Pokfulam Hong Kong SAR China; ^6^ Department of General Surgery (Colorectal Surgery) The Sixth Affiliated Hospital Sun Yat‐sen University Guangzhou China; ^7^ Li Ka Shing Institute of Health Sciences The Chinese University of Hong Kong Shatin Hong Kong SAR China; ^8^ Shenzhen Research Institute The Chinese University of Hong Kong Shenzhen China

**Keywords:** EGFR nanobody, oncomiR‐21, pancreatic cancer therapy, targeted RNA degradation

## Abstract

Pancreatic ductal adenocarcinoma (PDAC) remains a therapeutic challenge due to its dense stroma and lack of druggable targets. Through integrated bioinformatics analysis of in‐house and public datasets, we systematically identified miR‐21 as the most significantly upregulated oncomiR in PDAC, showing strong correlation with poor patient prognosis. RNA‐targeted degradation has emerged as a promising strategy for cancer treatment, enabling precise disruption of oncogenic signaling by degrading disease‐driving non‐coding RNAs. Nevertheless, poor tissue penetration and insufficient tumor specificity limit its therapeutic potential in pancreatic cancer, owing to the dense fibrotic stroma and heterogeneous target expression. Herein, we developed a dual‐targeting, bioresponsive Nb‐RIBOTAC (Nb‐Fc‐Val‐Cit‐RIBOTAC), engineered by conjugating a cathepsin B–responsive linker to bridge an EGFR‐targeting nanobody (Nb‐Fc fusion) with a miR‐21–specific RIBOTAC module. This rationally designed therapeutic achieved potent and selective miR‐21 degradation in orthotopic PDAC models, with 60% target knockdown while completely sparing normal tissues, leading to significant tumor growth inhibition. Our study establishes a transformative paradigm bridging bioinformatic identification with precision RNA degradation technology, offering new therapeutic possibilities for PDAC treatment. The target selection strategy and modular design principles described herein may be broadly applicable to other challenging malignancies.

## Graphical abstract

This study introduces an innovative strategy for the tumor‐selective catalytic degradation of oncogenic non‐coding RNA by interfacing a ribonuclease‐recruiting small molecule (RIBOTAC) with an EGFR‐targeting nanobody via a CTSB (Cathepsin B)‐responsive linker. Our Nb‐RIBOTAC platform enables precise delivery and activation of the RNA degrader within pancreatic cancer cells by leveraging both tumor‐associated antigen (EGFR) overexpression and the dysregulated protease activity in the tumor cells, achieving potent antitumor efficacy without systemic toxicity.

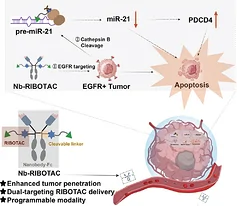

## 1 Introduction

Pancreatic ductal adenocarcinoma (PDAC) remains one of the most lethal malignancies, with a dismal 5‐year survival rate of less than 10% due to late diagnosis, dense desmoplastic stroma, immunosuppressive microenvironment, and high resistance to conventional therapies such as chemotherapy and immune checkpoint inhibitors (ICIs) [1]. The epidermal growth factor receptor (EGFR) signaling pathway is frequently dysregulated in pancreatic cancer, contributing to tumor proliferation, metastasis, and therapy resistance [2, 3, 4, 5]. However, direct targeting of EGFR with small‐molecule inhibitors or monoclonal antibodies has shown only modest clinical benefits, partly due to compensatory mechanisms and off‐target effects [6, 7, 8]. Thus, an alternative strategy that selectively degrades oncogenic RNA molecules downstream of EGFR signaling represents a more precise and effective therapeutic approach.

Through comprehensive bioinformatics analysis integrating data from in‐house and TCGA (The Cancer Genome Atlas), we identified microRNA‐21 (miR‐21) as a consistently overexpressed oncomiR not only in PDAC but across multiple gastrointestinal malignancies including colorectal and stomach adenocarcinoma. In PDAC specifically, miR‐21 emerges as a particularly critical regulator of tumor progression, with its overexpression strongly correlating with advanced disease stage and poor survival outcomes. Mechanistically, miR‐21 orchestrates a complex oncogenic network by simultaneously suppressing key tumor suppressors such as PTEN (Phosphatase and Tensin Homolog) and PDCD4 (Programmed Cell Death 4) while activating proliferative and anti‐apoptotic pathways, thereby driving tumor growth and conferring resistance to conventional therapies including gemcitabine‐based regimens [9, 10]. Despite its well‐established role in PDAC pathogenesis, therapeutic targeting of miR‐21 has remained elusive due to the inherent challenges of RNA‐directed drug development and the unique biological barriers presented by dense desmoplastic stroma [11, 12, 13, 14] [1]. Indeed, although RNA‐targeting strategies such as antisense oligonucleotides (ASOs) [15] and RNA interference (RNAi) [16, 17] have shown promise, their clinical translation has been hindered by poor tumor penetration, off‐target effects, and insufficient sustained RNA degradation in the fibrotic tumor microenvironment.

Recent advances in RNA‐targeting technologies, including ribonuclease‐targeting chimeras (RIBOTACs) [18, 19, 20, 21, 22, 23, 24, 25, 26], offer a promising avenue for degrading disease‐associated RNAs by recruiting endogenous RNases such as RNase L (Ribonuclease L). Nevertheless, conventional RIBOTACs suffer from limited tumor selectivity and bioavailability, as their small‐molecule scaffolds often lack efficient tissue penetration and cell‐specific delivery. Given that the mechanistic basis of RIBOTAC‐mediated RNA degradation has been well established by previous studies [24]^2^, our work instead focuses on addressing the delivery challenges in solid tumors by integrating EGFR‐targeted nanobody conjugation with a cathepsin B–responsive linker. We herein developed an EGFR‐targeted nanobody‐RIBOTAC (Nb‐RIBOTAC) conjugate, leveraging the high expression of EGFR in PDAC for tumor‐selective delivery while incorporating a protease‐cleavable Val‐Cit (Valine‐Citrulline) linker to enable cathepsin B‐responsive intracellular activation, thereby minimizing off‐tumor toxicity.

Our strategy builds upon emerging antibody‐guided RNA degradation platforms, such as Aptamer‐RIBOTAC (ARIBOTAC) [26] and inducible RIBOTAC (iRIBOTAC) [23], which enhance precision through tumor‐specific activation. By integrating bioinformatics validation of miR‐21's oncogenic role with nanobody‐mediated targeting, we demonstrate that Nb‐RIBOTAC achieves >60% miR‐21 knockdown in EGFR‐high PDAC models, restoring tumor suppressor pathways and inducing selective cytotoxicity. This approach not only overcomes stromal barriers but also minimizes systemic toxicity, representing a transformative paradigm for RNA‐targeted therapy in PDAC and other solid tumors. Here, we present the first proof‐of‐concept study combining EGFR‐directed nanobody delivery with enzyme‐responsive RNA degradation, offering a novel solution to the undruggability of non‐coding oncogenic RNAs. Collectively, our findings highlight the potential of RIBOTAC technology to bridge RNA biology and precision oncology, paving the way for clinical translation in aggressive malignancies.

## 2 Results

### 2.1 Pan‐GI Analysis Prioritized miR‐21 as a Promising Therapeutic Target in PDAC

To prioritize suitable RNA targets for degradation, we performed systematic analysis of miRNA expression profiles across multiple gastrointestinal (GI) cancers. We employed three in‐house cohorts, including colorectal cancer (CRC), pancreatic ductal adenocarcinoma (PDAC), and esophageal squamous cell carcinoma (ESCC), and a stomach adenocarcinoma (STAD) cohort from The Cancer Genome Atlas (TCGA) [27]. The sample sizes were as follows: CRC (99 normal vs. 100 tumor) (Table S1), ESCC (155 normal vs. 155 tumor) (Table S2), PDAC (60 normal vs. 59 tumor) (Table S3), and STAD (45 normal vs. 446 tumor) (Table S4). Given their shared anatomical and biological features, these four cancers were analyzed collectively as a pan‐GI cohort. Differential expression analysis identified 40, 88, 119, and 133 miRNAs significantly upregulated (Benjamini‐Hochberg adjusted *p* <0.05, log_2_ fold‐change >1) in CRC, PDAC, ESCC, and STAD, respectively, compared to tumor‐adjacent normals (Tables S1–S4). Among all the differential miRNAs, six candidates showed consistent upregulation in all four cancer types (Figure 1a,b).

**FIGURE 1:**
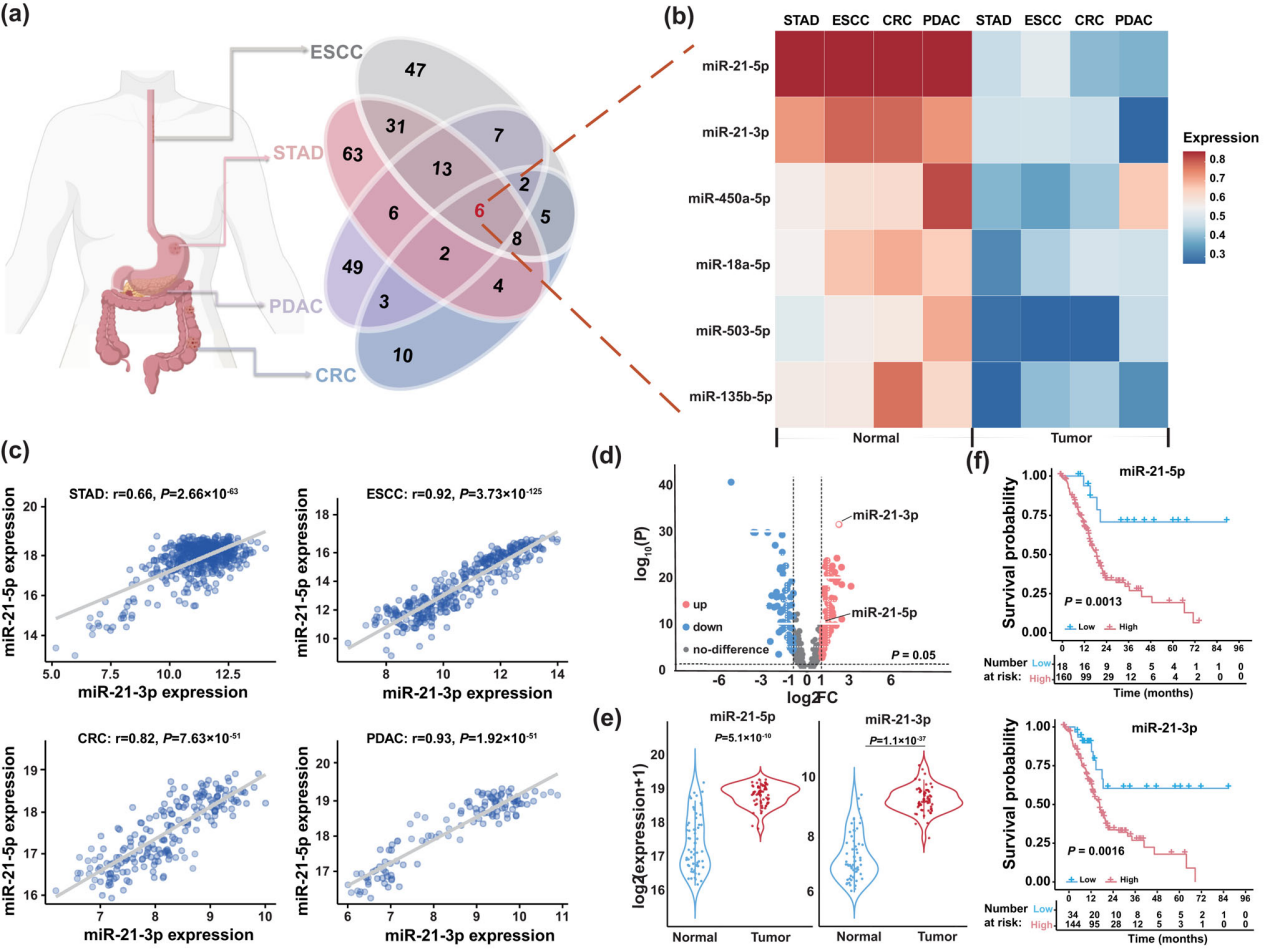
Comprehensive bioinformatic analysis identified miR‐21 as a promising therapeutic target in pancreatic cancer and other major GI malignancies. (a) Venn diagram illustrates the overlap of significantly upregulated miRNAs (adjusted *p* < 0.05, log_2_FC >1) among four gastrointestinal cancers: esophageal squamous cell carcinoma (ESCC, n = 119), stomach adenocarcinoma (STAD, n = 133), pancreatic ductal adenocarcinoma (PDAC, n = 88), and colorectal cancer (CRC, n = 40). (b) Hierarchical clustering heatmap demonstrates the expression profile of six concurrently up‐regulated miRNAs in four cancer cells. (c) The correlation between miR‐21‐5p and miR‐21‐3p expression in four gastrointestinal cancers. (d) Volcano plot displays the differential expression pattern of miRNAs in PDAC versus adjacent normal tissues. miR‐21‐3p (highlighted in red) shows the most dramatic upregulation. *p*‐values were calculated based on a negative binomial generalized linear model (Wald test) implemented in the DESeq2 R package. (e) Violin plot compares miR‐21‐3p and mir‐21‐5p expression levels between PDAC tissues and adjacent normal tissues. *p*‐values were calculated based on a negative binomial generalized linear model (Wald test) implemented in the DESeq2 R package. (f) Kaplan–Meier survival curve analyzes the association between miR‐21 (miR‐21‐3p and mir‐21‐5p) expression and patient prognosis in PDAC (TCGA dataset). *p*‐values were calculated based on Pearson correlation tests.

Notably, both the mature strands, miR‐21‐5p and miR‐21‐3p, were significantly upregulated across all four cancer types. As both are processed from the same pre‐miR‐21 precursor, this coordinated upregulation suggests that dysregulation occurs at the level of the precursor transcript.

To further assess this possibility, we evaluated the correlation between miR‐21‐5p and miR‐21‐3p expression. As expected, their expression levels were indeed strongly positively correlated in each cancer type (Figure 1c, STAD: r = 0.66, *p* = 2.66 × 10^−63^; ESCC: r = 0.92, *p* = 3.73 × 10^−125^; CRC: r = 0.82, *p* = 7.63 × 10^−51^; PDAC: r = 0.93, *p* = 1.92 × 10^−51^), indicating that their abundance is likely co‐regulated by the production of their common precursor. Moreover, we performed external validations using four independent public GEO datasets encompassing major gastrointestinal cancers, including colorectal cancer (GSE115513, CRC), gastric cancer (GSE23739, GC), pancreatic cancer (GSE119794, PDAC), and esophageal cancer (GSE13937, ESCC). Across these validation cohorts, both miR‐21‐3p and miR‐21‐5p were generally elevated in tumor tissues relative to paired adjacent normal tissues (Figure S8). These results further support that miR‐21 upregulation is a robust and reproducible feature across gastrointestinal cancers.

The persistent dysregulation of miR‐21 in PDAC contributes to tumor proliferation, invasion, chemoresistance, and poor prognosis by suppressing key tumor suppressors such as PTEN, PDCD4, and RECK, while promoting oncogenic pathways like PI3K/AKT and Bcl‐2‐mediated anti‐apoptosis [28, 29]. miR‐21‐3p ranks top among the upregulated miRNAs (Figure 1d), while both miR‐21 strands showed significant upregulation in PDAC (Figure 1e). We next evaluated the prognostic value of both miR‐21‐5p and miR‐21‐3p using TCGA survival data. Kaplan–Meier analysis demonstrated that high expression of both miR‐21‐3p (*p* = 0.0015) and miR‐21‐5p (*p* = 0.0013) was significantly associated with poor overall survival (Figure 1f). Taken together, our findings suggest that miR‐21 may play a pivotal role in PDAC and other GI cancers.

Given that both oncogenic strands are derived from the same precursor, we selected pre‐miR‐21 as the primary RNA target for our Nb‐RIBOTAC‐based degradation strategy (Scheme 1). This approach aims to exploit the catalytic efficiency of RNase‐mediated RNA degradation while leveraging EGFR‐targeted nanobodies for tumor‐selective delivery, offering a novel therapeutic avenue against this lethal disease.

**SCHEME 1:**
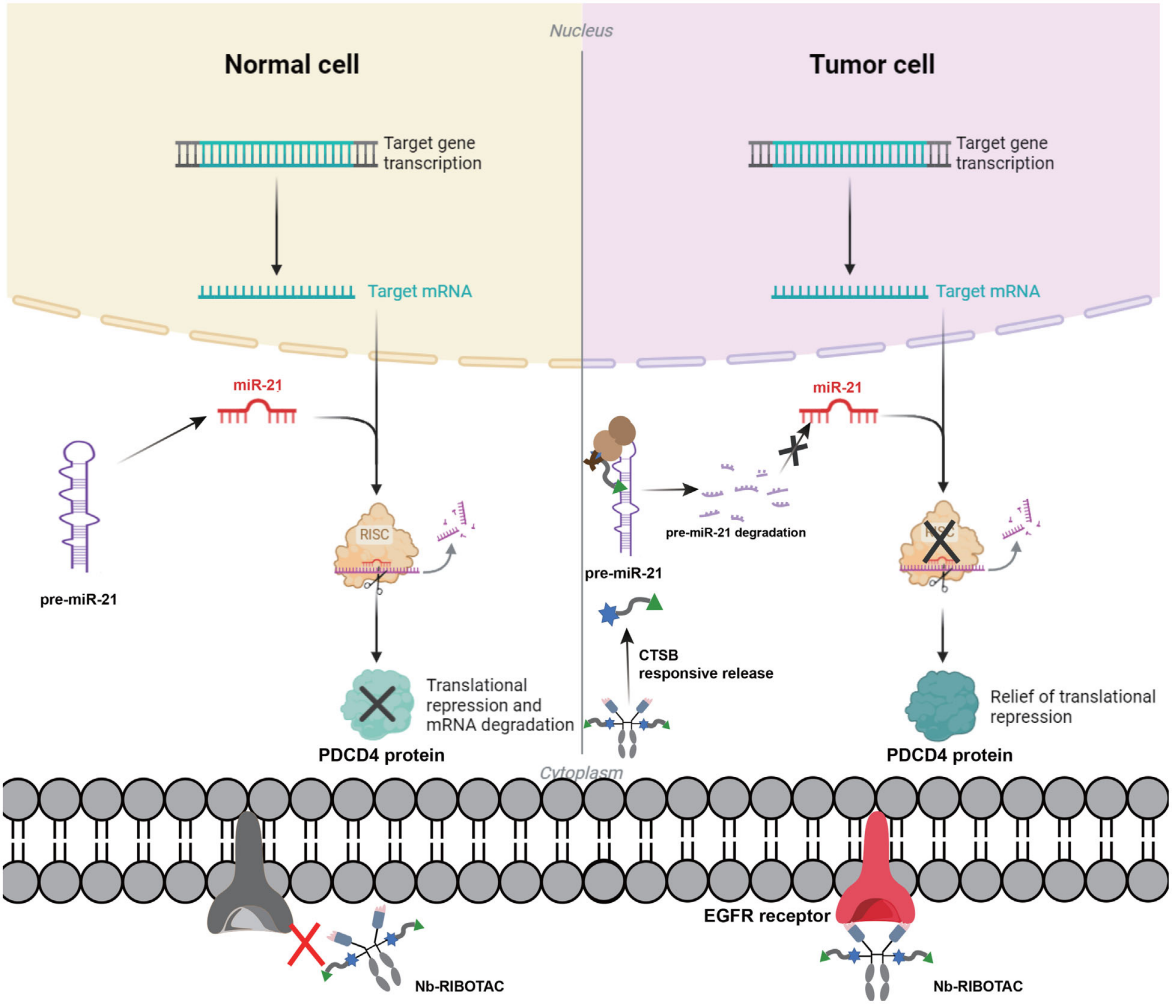
Scheme illustration of Nb‐RIBOTAC‐Mediated targeted degradation of oncogenic miR‐21 in cancer cells.

### 2.2 Design and Optimization of miR‐21 Degrader

To achieve targeted degradation of oncogenic miR‐21 in PDAC, we employed the RIBOTAC platform, which enables catalytic RNA degradation by recruiting endogenous RNase L to the target transcript [, 21]. While traditional RIBOTACs offer a powerful mechanism for RNA depletion, their clinical translation has been hindered by several limitations, including high molecular weight, poor hydrophilicity, weak tissue penetration, and lack of tumor selectivity, particularly problematic in PDAC due to its dense, fibrotic stroma that restricts macromolecule diffusion.

To overcome these challenges, we pursued an antibody‐guided RIBOTAC strategy, leveraging the advantages of nanobodies (Nbs) [30, 31, 32] for enhanced tumor penetration and EGFR‐specific targeting. Nanobodies (∼15 kDa) exhibit superior stromal penetration compared to full‐length antibodies (∼150 kDa) while retaining high binding affinity and specificity. We engineered a bispecific Nb‐RIBOTAC conjugate composed of an anti‐EGFR nanobody (Nb‐EGFR) (Figure 2a) for selective delivery to EGFR‐overexpressing PDAC cells and a RIBOTAC [33] module targeting miR‐21 (Figure 2b), designed with an optimized RNA‐binding motif complementary to the miR‐21 stem‐loop structure.

**FIGURE 2:**
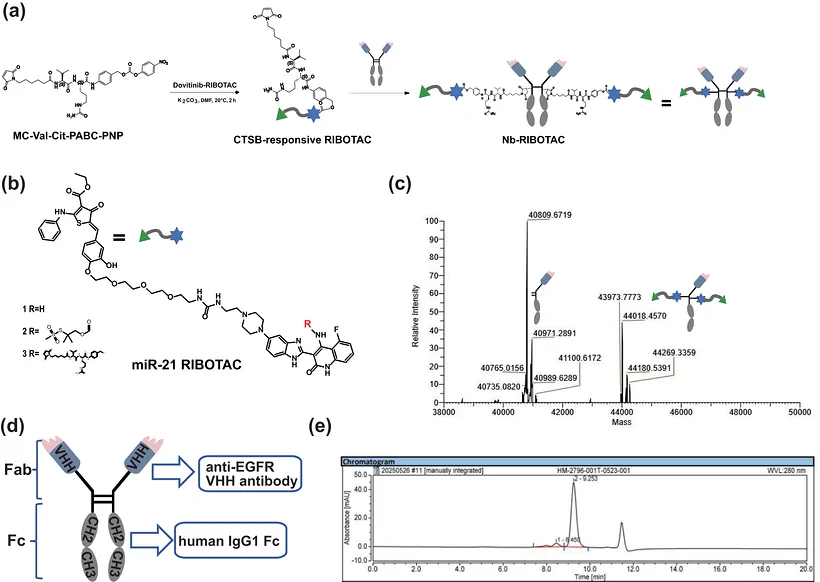
Design and optimization of Nb‐RIBOTAC. (a) Synthesis route of Nb‐RIBOTAC. (b) Structure of miR‐21 RIBOTAC. (c) Mass spectrometric characterization of Nb‐RIBOTAC. (d) Structure of EGFR nanobody fused with human IgG1 Fc. (e) Purification profile of Nb‐RIBOTAC.

Linker optimization for tumor‐specific activation. To ensure precise RIBOTAC release within tumors while minimizing off‐target effects, we evaluated two tumor microenvironment‐activated linkers: a GSH (Glutathione)‐responsive linker [34] (Figure 2b, Figures S1–S3) and Val‐Cit linker [35] (Figure 2b, Figures S4–S6), which are cleaved by cathepsin B, a protease overexpressed in PDAC lysosomes. Initial conjugation attempts revealed that conventional nanobodies exhibited low binding affinity for the RIBOTAC payload, likely due to steric hindrance. To address this, we fused the Nb‐EGFR with a human Fc domain (human IgG1 Fc) (Figure 2c,d and Table S5), which prolonged serum half‐life and improved conjugate efficiency. The characterization of the Nb‐RIBOTAC conjugate by reversed‐phase mass spectrometry (RP‐MS) (Figure 2c) and final purity 92.11% (Figure 2e, Table S6 and Figure S8). Given the intrinsically high molecular weight and strong hydrophobicity of small‐molecule degraders such as PROTACs (Proteolysis‐Targeting Chimeras) and RIBOTAC, their conjugation to antibodies—particularly VHH(Variable domain of Heavy chain)‐Fc fusions, which exhibit lower hydrophilicity than conventional antibodies—has remained technically challenging. Although conjugation of PROTACs to traditional antibodies has been previously reported, [35, 36, 37], successful coupling of VHH‐Fc with RIBOTAC has not been described to date. Through extensive optimization of conjugation conditions, we achieved, for the first time, the successful conjugation of a VHH‐Fc scaffold with a riboTAC, representing the first example of a degrader conjugate based on a smaller antibody format (VHH‐Fc), to our knowledge. Notably, we observed that a drug‐to‐antibody ratio (DAR) of 1.4 led to severe aggregation and precipitation, whereas optimization enabled a stable DAR of 1.23, highlighting the critical balance between payload hydrophobicity and conjugate stability in this novel format. Moreover, upon conjugation of the small molecule at a DAR of 1.23 as calculated by Figure 2c, the half‐maximal effective concentration (EC_50_) for Fc functional activity shifted from 29.33 to 54.38 nm, while the binding affinity (KD) determined by Surface Plasmon Resonance (SPR) analysis increased from 1.16 to 2.28 nm, corresponding to an approximately twofold reduction in potency (Figure S9). This modest shift is consistent with partial steric hindrance and reduced valency of functional Fc domains following conjugation and remains within an acceptable range for retention of effector function. Through systematic optimization, the Fc‐fused nanobody platform conjugated with RIBOTAC via a Val‐Cit linker emerged as the superior strategy, demonstrating multiple critical advantages over conventional formats. This engineered construction exhibited dramatically improved conjugation efficiency, achieving a 15.7‐fold increase in successful payload attachment compared to plain nanobody conjugates, as quantified by HPLC peak integration. Importantly, while benefiting from Fc‐mediated neonatal receptor recycling, the compact nanobody component maintained superior tumor penetration capabilities. The Val‐Cit linker chemistry proved essential for precise intracellular activation. To validate the cathepsin B‐responsive cleavage of the Val‐Cit linker, we performed an in vitro cleavage assay (Figure S10). The reaction mixture was sampled at 0, 3, 6, and 8 h, and the cleavage products were analyzed by HPLC. The intact conjugate was progressively cleaved over time, with approximately 30% cleavage observed at 3 h, near‐complete cleavage achieved by 6 h, and full cleavage confirmed at 8 h. To confirm that this cleavage is specifically mediated by cathepsin B, a parallel reaction was conducted in the presence of the cathepsin B inhibitor CA‐074. At the 8 h time point, cleavage was substantially suppressed, demonstrating that the observed linker cleavage is indeed dependent on cathepsin B activity. Collectively, these results validate the efficient and selective responsiveness of the Val‐Cit linker to cathepsin B, supporting its utility for enzyme‐triggered payload release. This comprehensive optimization resulted in a therapeutic candidate that successfully reconciles the typically conflicting requirements of prolonged circulation, deep tumor penetration, and cathepsin B‐driven intracellular release—key challenges that have historically limited the efficacy of targeted RNA therapeutics in solid tumors. The final construct Nb‐Fc‐Val‐Cit‐RIBOTAC, shortly named Nb‐RIBOTAC, demonstrated dual‐targeting functionality: EGFR‐mediated cellular uptake and tumor‐specific RIBOTAC release. Intracellular cathepsin B cleaves the Val‐Cit linker, liberating the active RIBOTAC to recruit RNase L and degrade miR‐21 in tumor cells and remaining inactivated in normal cells.

### 2.3 Nb‐RIBOTAC Induced Selective Degradation of miR‐21 in Specific Cancer Cells

To validate the EGFR‐dependent targeting specificity and therapeutic efficacy of Nb‐RIBOTAC (Figure 3a), we first characterized EGFR expression profiles across pancreatic cancer cell lines (including PANC‐1, ASPC‐1, S2VP10, PK‐8, MIA PaCa‐2, KLM) and normal cells (HPNE and MCF‐10A) using western blot analysis (Figure 3b and Figure S11). The results revealed markedly elevated EGFR protein levels in PANC‐1, ASPC‐1, and S2VP10 cells compared to PK‐8, MIA PaCa‐2, KLM cells and normal HPNE and MCF‐10A cells, establishing a cellular model system with differential EGFR expression for subsequent experiments (Figure 3c). As shown in Figure 3d,e, the treatment of PANC‐1 cells with Nb‐RIBOTAC caused comparable miR‐21 degradation to that of free RIBOTAC treatment.

**FIGURE 3:**
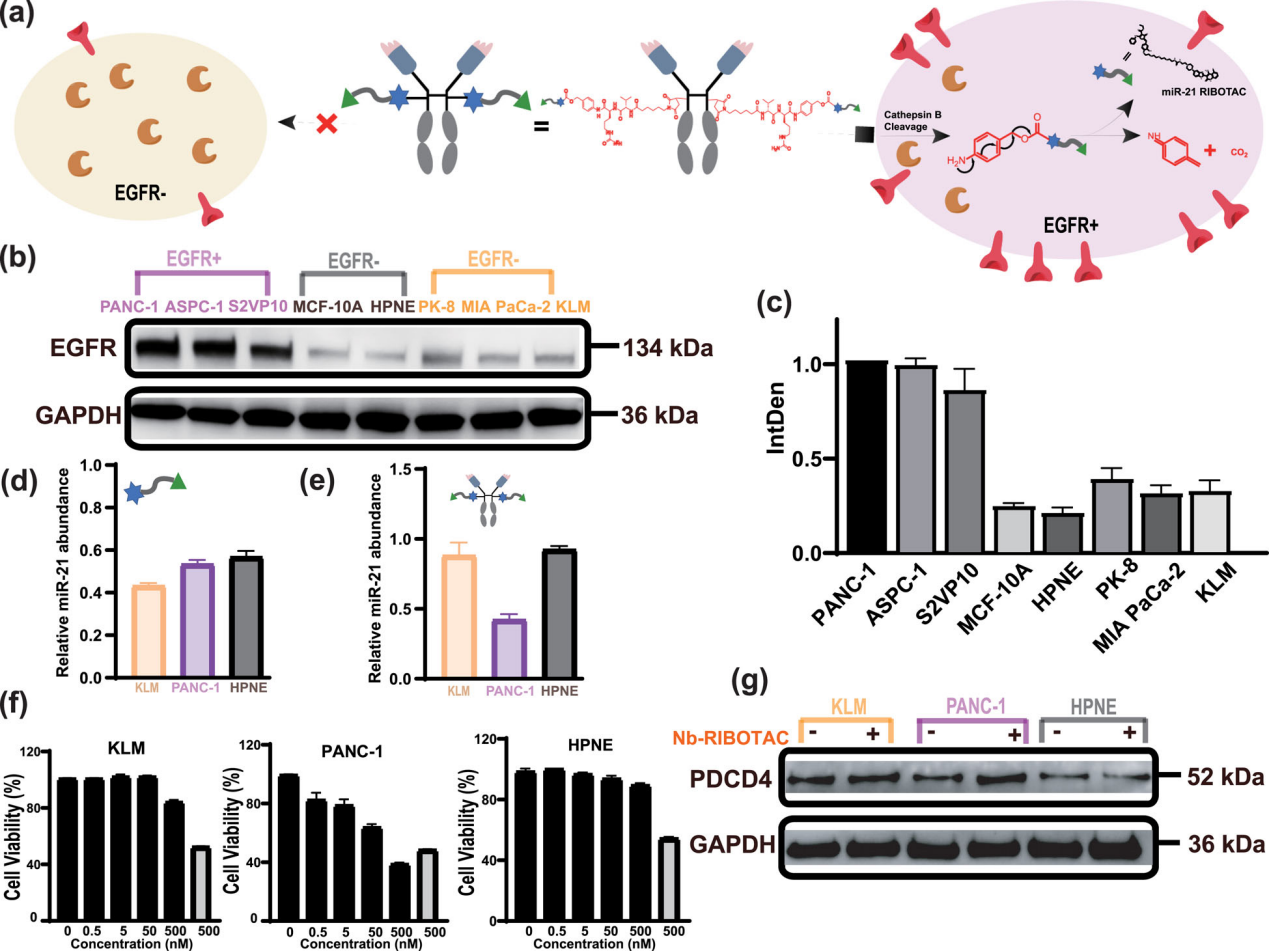
Evaluation of the tumor‐targeting capability of Nb‐RIBOTAC. (a) Illustration of the tumor‐specific activation process of CTSB‐responsive Nb‐RIBOTAC. (b) Western blot analysis of EGFR levels in six PDAC cancer cell lines (PANC‐1, ASPC‐1, S2VP10, PK‐8, MIA PaCa‐2, KLM) and two normal cell lines (HPNE and MCF‐10A). (c) Quantitative analysis of EGFR expression (n = 3). (d) qPCR results of miR‐21 levels in PANC‐1, KLM and HPNE treated with miR‐21 RIBOTAC and (e) Nb‐RIBOTAC (n = 3). (f) Dose dependence of Nb‐RIBOTAC treated with PANC‐1, KLM and HPNE cells for 48 h (n = 3). (g) Western blot analysis of PDCD4 protein levels treated with or without Nb‐RIBOTAC in PANC‐1, KLM and HPNE cells (n = 3).

Treatment with Nb‐RIBOTAC induced profound miR‐21 degradation (>60% reduction by RT‐qPCR) specifically in EGFR‐high cancer cells, while showing minimal effects in EGFR‐low cancer cells and normal pancreatic cells (Figure 3d). To confirm that the observed miR‐21 knockdown is mediated by RNase L, we performed parallel experiments in the presence of an RNase L inhibitor. As shown in Figure S12, RNase L inhibition effectively blocked miR‐21 degradation, confirming that the RIBOTAC mechanism operates through RNase L recruitment. To further validate that the therapeutic effects are driven by EGFR‐specific delivery, we conducted EGFR blocking experiments. Pre‐incubation with an excess of Nb substantially attenuated miR‐21 knockdown in EGFR‐positive cells (Figure S12), confirming the EGFR‐dependent activity of the Nb‐RIBOTAC conjugate. Functional characterization through CCK‐8 (Cell Counting Kit‐8) viability tests (Figure 3f) revealed that the selective miR‐21 degradation translated to potent anti‐tumor effects exclusively in EGFR‐high cell lines. The treatment resulted in a dramatic 60% reduction in proliferating cells, while exhibiting negligible impact on the viability of EGFR‐low KLM cells and normal HPNE cells. Moreover, mechanistic investigations confirmed that the observed selective cytotoxicity was mediated through restoration of the PDCD4 expression signaling pathway specifically in EGFR‐high cancer cells (Figure 3g and Figure S13), suggesting the biocompatibility and minimal negative effect of PDCD4 regulation and apoptosis induction of Nb‐RIBOTAC in cancer cells.

### 2.4 Cellular Internalization and Pathway Mechanism of Nb‐RIBOTAC

To investigate the cellular internalization dynamics and further analyze the fate of Nb‐RIBOTAC within the intracellular environment, we performed time‐course tracking studies using AF647‐labeled Nb‐RIBOTAC by confocal microscopy. The Fc domain was site‐specifically conjugated with AF647 fluorophore through NHS ester chemistry targeting free amino groups, ensuring minimal interference with the nanobody's binding capability. EGFR+ PANC‐1 cells were incubated with 100 nm AF647‐labeled Nb‐RIBOTAC for 1 h, and cells were then washed and imaged at 1, 4, 8, and 24 h time points to study conjugate trafficking (Figure 4a). At 1 h, AF647‐labeled Nb‐RIBOTAC was observed on the cell surface, whereas after 4 h of incubation, AF647‐labeled Nb‐RIBOTAC was already partially internalized into distinct intracellular compartments, presumably early and late endosomes upstream from lysosome trafficking for degradation, and partially colocalized with LysoTracker Green‐labeled lysosomes (Figure 4a,c). Confocal microscopy analysis revealed a time‐dependent increase in cellular uptake, with punctate fluorescent signals becoming detectable at 1‐h post‐treatment and progressively intensifying over the 2–4‐h timepoints (Figure 4a). Quantitative image analysis demonstrated complete internalization by 6 h, as evidenced by the disappearance of membrane‐associated fluorescence and exclusive cytoplasmic localization. Note that, upon lysosomal digestion of Nb‐RIBOTAC, the free active RIBOTAC must be released and then trigger miR‐21 degradation. HPNE cells expressing very low levels of EGFR receptor were unable to uptake AF647‐labeled Nb‐RIBOTAC under the same conditions (Figure 4b), consistent with resistance of these cells to Nb‐RIBOTAC‐mediated miR‐21 degradation. EGFR‐positive cells exhibited clear membrane‐associated signal at this early point, whereas no specific binding was observed in EGFR‐negative cells, confirming the selectivity of the Nb‐RIBOTAC conjugate.

**FIGURE 4:**
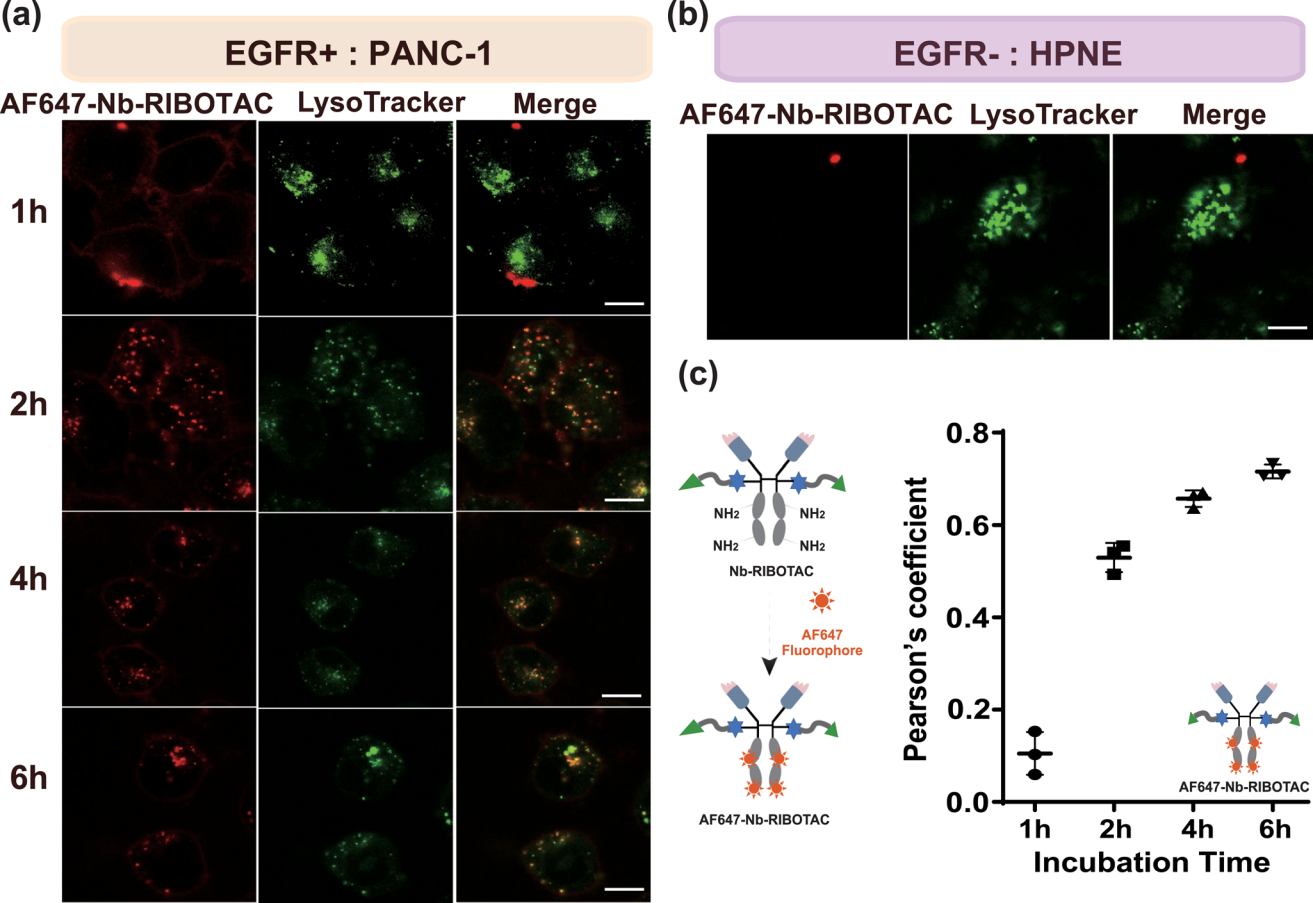
In vitro internalization study of Nb‐RIBOTAC. (a) EGFR+ PANC‐1 cells were treated with AF647‐conjugated Nb‐RIBOTAC (100 nM) for 1, 2, 4, and 6 h (all post‐1‐h washout). Cells were then labeled with LysoTracker Green (50 nm, 30 min). Shown is a representative frame from live imaging of cells via confocal microscopy. Scale bars = 20 µm. (b) EGFR− HPNE cells were treated with Nb‐RIBOTAC (100 nm) for 30 min, followed by incubation with LysoTracker Green (50 nm) for an additional 30 min. Shown is a representative frame from live imaging of cells via confocal microscopy. (c) Quantification of AF647‐conjugated Nb‐RIBOTAC colocalization with LysoTracker Green. Pearson's correlation coefficient is shown for various incubation time points in PANC‐1 cells; n = 3 cells.

Colocalization studies with LysoTracker Green provided crucial mechanistic insights into the endocytic pathway. The AF647 signal initially showed partial overlap with early endosome markers at 1–2 h, followed by progressive accumulation in lysosomal compartments. By 6 h, >70% of the AF647 signal colocalized with lysosomal markers, indicating complete internalization and trafficking to degradative organelles. Control experiments using AF647‐labeled non‐targeting IgG confirmed the specificity of this uptake pattern (Figure S14). To quantitatively analyze the intracellular trafficking dynamics, we performed Pearson's correlation coefficient (PCC) analysis of the AF647‐labeled Nb‐RIBOTAC with LysoTracker Green across different time points. The PCC values demonstrated a time‐dependent increase in lysosomal colocalization, rising from 0.1 at 1 h to 0.7 at 6 h post‐treatment (Figure 4c). These quantitative colocalization metrics, combined with the onset of miR‐21 degradation (Figure 3d). (detected by RT‐qPCR), provide robust statistical support for our lysosome‐activated delivery strategy. These findings provide direct visual evidence that our Nb‐RIBOTAC conjugate undergoes efficient and specific EGFR‐mediated internalization, with complete lysosomal delivery occurring within 6 h, a kinetic profile that aligns optimally with the designed protease‐activated degradation mechanism. Further, by using RNA‐seq analysis, we observed coordinated downregulation of key lipid metabolic genes (SCD, FADS, SORBS1, PLIN4, ACSL5, ACOXL) through transcriptomic analysis (Figure S15 and Table S7), with KEGG pathway enrichment specifically pointing to suppressed PPAR signaling (Figure S16 and Table S8). This suggests that miR‐21 degradation may derepress upstream suppressors of the PPAR pathway, leading to broad inhibition of lipid synthesis, storage, and oxidation. Beyond direct oncogene modulation, this metabolic reprogramming disrupts cancer cell energy homeostasis and membrane integrity, potentially synergizing with EGFR targeting to suppress tumor growth, overcome drug resistance, and inhibit metastasis through dual‐pathway intervention.

### 2.5 Nb‐RIBOTAC Exhibits Improved In Vivo Antitumor Activity in PANC‐1 Xenograft Mouse Model

The formulation of RIBOTAC with Nb‐Fc‐EGFR can not only enhance the aqueous solubility of RIBOTAC but also improve the accumulation of RIBOTAC in tumors by making use of the targeting efficiency and superior tumor penetration capabilities of Nb‐Fc‐EGFR. Herein, the in vivo delivery of Nb‐RIBOTAC for apoptosis induction and potential cancer therapy was studied using PANC‐1 tumor‐bearing mouse xenograft.

For evaluating in vivo distribution and tumor‐targeted delivery efficacy, Nb‐RIBOTAC was labeled with fluorescent AF647. The AF647‐labeled Nb‐RIBOTAC was administered intravenously to PANC‐1 tumor‐bearing mice at a protein dosage of 2.5 mg/kg at 3, 6, 24 and 48h. After 48 h, the mice were imaged and then sacrificed, and further their tissues were harvested for ex vivo fluorescence imaging. The distinct biodistribution profile, characterized by rapid tumor accumulation evident in ex vivo tissues as early as 3 h and ultimately predominant tumor‐specific localization by 48 h (Figure 5a), provides critical pharmacodynamic justification for a Q48h (every 48‐h) dosing strategy.

**FIGURE 5:**
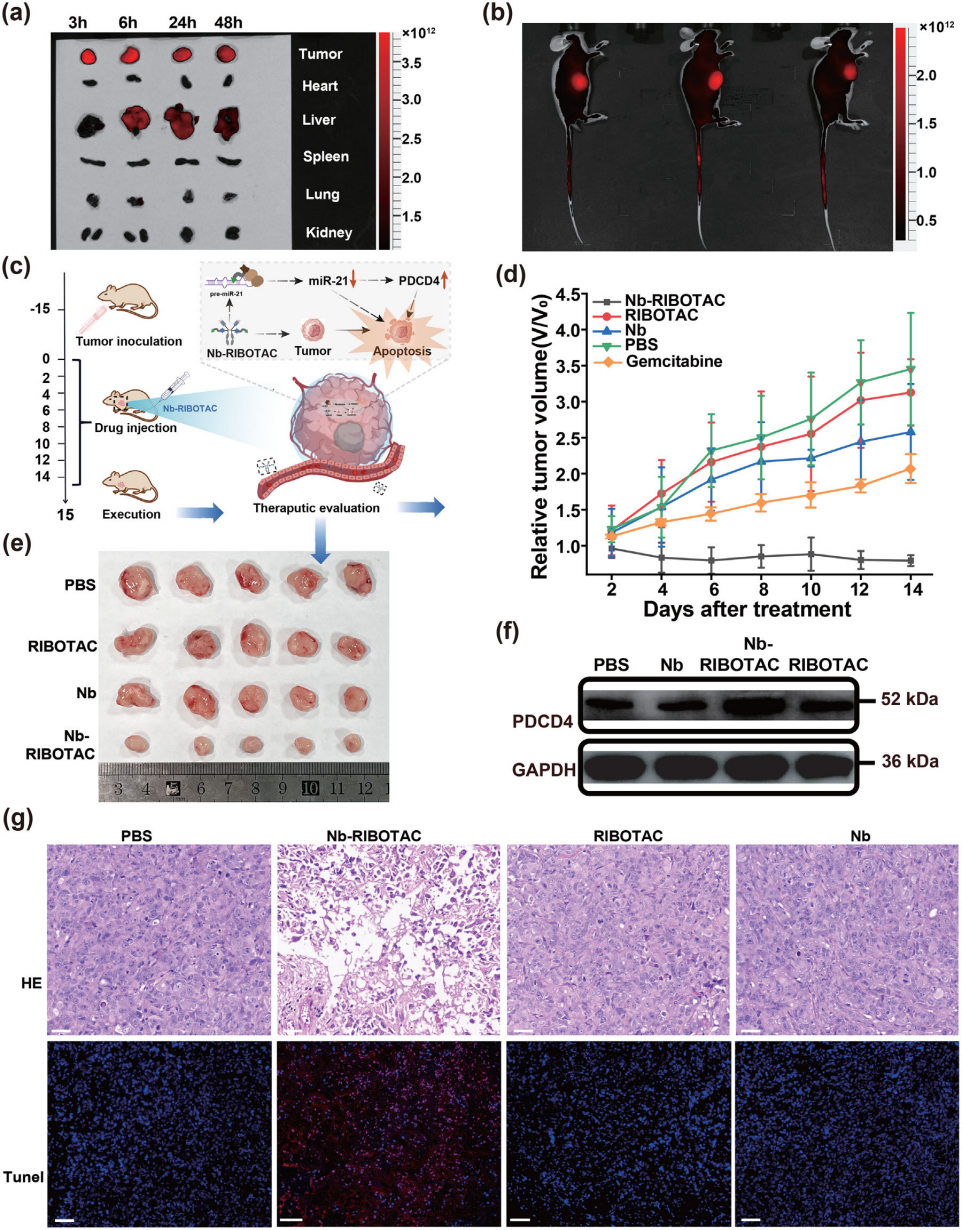
Antitumor effect of Nb‐RIBOTAC in PANC‐1 xenograft mouse models. (a) Biodistribution of Nb‐RIBOTAC in PANC‐1 tumor‐bearing Nu/Nu nude mice through vein tail injection. Nb‐RIBOTAC was labeled by AF647 for fluorescent imaging. (b) In vivo fluorescence imaging of Nb‐RIBOTAC in PANC‐1 xenograft models at 48h (n = 3). (c) Schematics of in vivo antitumor study of Nb‐RIBOTAC. (d) Tumor growth curves of tumor‐bearing mice are subject to diverse treatments. Data represented as mean ± s.d. (n =  5). (e) Tumor autopsy results of different groups of mice at the end of the experiment (n = 5 mice/group). (f) Western blotting detection of PDCD4 level in tumors after Nb‐RIBOTAC treatment and other negative controls (n =  5). (g) H&E staining and TUNEL staining (for apoptosis) of tumors in mice that received different treatments as indicated. Scale bars: 200 µm.

This spatiotemporal pattern demonstrates that while the nanobody moiety facilitates immediate target engagement, the Fc domain confers sufficient plasma persistence to overcome initial non‐specific MPS uptake and allow for comprehensive background clearance. The resultant high tumor‐to‐background ratio achieved at 48 h (Figure 5b and Figure S17) indicates not only optimal imaging contrast but, more importantly, confirms that therapeutic target saturation is maintained at this interval. Consequently, the robust and specific tumor signal at 48 h serves as a direct biomarker for effective dosing timing. Furthermore, to directly address whether Fc fusion compromises tumor penetration, we performed a head‐to‐head comparison between our VHH‐Fc (∼80 kDa) and a full‐length antibody (cetuximab, ∼150 kDa) in the same PANC‐1 mouse tumor model with Matrigel. The smaller VHH‐Fc rapidly penetrated this dense stromal barrier and enriched in tumors within 3 h, while the full‐size antibody was excluded and consequently cleared by the liver (Figure S18). Collectively, these findings validate that the compact VHH‐Fc format is critical for overcoming the desmoplastic barrier in PDAC, thereby ensuring efficient tumor accumulation and supporting the rationale for using nanobody‐based delivery in our RIBOTAC conjugate design.

This compelling pharmacokinetic evidence forms the basis for adopting a Q48h regimen in subsequent efficacy studies. Readministration at this 48‐h window is strategically timed to reinforce tumor drug levels before significant decline, ensuring continuous target coverage and maximizing therapeutic effect while leveraging the minimized systemic exposure to mitigate potential cumulative toxicity. In the evaluation of the antitumor efficacy of the Nb‐RIBOTAC delivery, PANC‐1 tumor‐bearing mice were administered with the Nb‐RIBOTAC at a dosage of 20 mg/kg, along with phosphate‐buffered saline (PBS), Nb‐Fc‐EGFR, and

RIBOTAC alone as negative controls, and gemcitabine (60 mg/kg) as a positive control (Figure 5c). We found that the administration of Nb‐RIBOTAC effectively suppressed tumor growth down to 20% of PBS‐administrated controls (Figure 5d,e and Figure S19). In contrast, the administration of Nb‐Fc‐EGFR and RIBOTAC alone did not show an effective anti‐tumor effect. Western blot analysis of tumoral PDCD4 indicated that Nb‐RIBOTAC administration indeed increases PDCD4 in tumors compared to that of negative controls (Figure 5f and Figure S20), showing a higher selectivity toward the degradation of miR‐21. Nb‐RIBOTAC did not induce significant weight changes following drug administration (Figure S21). Histological examinations, including hematoxylin and eosin (H&E) staining and apoptosis assay using TUNEL staining of tumor sections revealed substantial cell apoptosis in the tumors of mice treated with Nb‐RIBOTAC nanoparticles (Figure 5g), while minimal apoptosis was observed in negative controls. Mice weight monitoring (Figure S21) and H&E staining of major organs (Figure S22) further confirmed the biocompatibility of Nb‐RIBOTAC nanoparticles for in vivo protein delivery. Additionally, liver function analysis indicated minimal hepatocellular injury following the in vivo delivery of Nb‐RIBOTAC, as evidenced by minimal changes in aspartate transaminase (AST), alanine aminotransferase (ALT), and total bilirubin levels in the serum of mice receiving the mentioned injections (Figure S14). Altogether, both tumor growth suppression and biocompatibility studies indicated the advantages of in vivo delivery of Nb‐RIBOTAC to regulate miRNA for potential cancer therapy. As a positive control, gemcitabine treatment led to moderate tumor growth inhibition (Figure 5d and Figure S19), with efficacy intermediate between the PBS control and the Nb‐RIBOTAC group but was substantially less effective than Nb‐RIBOTAC. Despite this antitumor activity, gemcitabine induced pronounced systemic toxicity, evidenced by significant body weight loss (Figure S20) and marked elevations in serum creatinine (CREA) and urea (UREA) levels, both exceeding two‐fold increases relative to controls (Figure S23). While AST levels remained unchanged, ALT activity decreased by more than 50%, a finding likely attributable to gemcitabine‐induced cachexia rather than direct hepatocellular injury. In contrast, mice treated with Nb‐RIBOTAC showed no significant changes in body weight, CREA, UREA, or transaminase levels throughout the study. The absence of toxicity markers, combined with superior antitumor efficacy, highlights the favorable therapeutic index of the Nb‐RIBOTAC conjugate. This targeted delivery strategy thus minimizes systemic exposure of the RIBOTAC payload, mitigating the off‐target toxicity associated with untargeted agents such as gemcitabine while achieving enhanced tumor suppression.

## 3 Discussion

We initially identified miR‐21 as a broadly overexpressed oncomiR across multiple gastrointestinal cancers, with its high expression particularly correlated with poor prognosis in pancreatic ductal adenocarcinoma (PDAC). This compelling association established miR‐21 as a promising therapeutic target for this aggressive malignancy. In this proof‐of‐concept study, we presented a targeted RNA degradation strategy that integrates EGFR‐specific delivery with enzyme‐responsive activation for the selective degradation of oncogenic miR‐21 in PDAC. The choice of an EGFR‐targeting nanobody as the delivery module is particularly advantageous for PDAC, given its ability to penetrate deeply into the dense, fibrotic tumor stroma, which limits the efficacy of larger monoclonal antibodies. The designed Nb‐Fc‐Val‐Cit‐RIBOTAC conjugate thus leverages this superior tumor penetration capability for efficient accumulation, while its Val‐Cit linker ensures proteolytically triggered release of the RNA‐degrading payload specifically within the tumor stroma. This dual‐level targeting—cellular via the nanobody and subcellular via the CTSB‐cleavable linker—enables potent miR‐21 knockdown, suppression of tumor growth, and reactivation of tumor suppressor pathways across multiple PDAC models.

To establish a model system for evaluating EGFR‐dependent targeting, we first screened a panel of PDAC cell lines and selected pairs with high and low EGFR expressions. Using these models, we demonstrated that the cytotoxic effect of Nb‐RIBOTAC was significantly more pronounced in high‐EGFR cells. Furthermore, qPCR and western blot analyses confirmed that Nb‐RIBOTAC‐mediated miR‐21 degradation led to a subsequent upregulation of the tumor suppressor PDCD4 in a manner dependent on EGFR expression levels, providing direct functional evidence of its selective delivery and on‐target activity. The 500 nM concentration used in our in vitro assays was selected based on dose–response curves (Figure 3f), which identified this condition as optimal for achieving robust miR‐21 knockdown while minimizing nonspecific cytotoxicity. Notably, the specificity of Nb‐RIBOTAC for EGFR‐positive cells creates a favorable therapeutic window, as it selectively induces cytotoxicity in EGFR‐high tumor cells while sparing normal cells (Figure 3d), in contrast to the non‐targeted RIBOTAC, which exhibits comparable toxicity to both cancer and normal cells. PDCD4 was selected as a downstream marker based on extensive literature establishing it as a direct transcriptional target of miR‐21 with well‐documented pro‐apoptotic functions [38, 39]. Notably, PDCD4 restoration has been shown to actively contribute to apoptosis induction, rather than merely serving as a secondary consequence of cell death [40]. Within the RIBOTAC field, PDCD4 has been consistently employed as a pharmacodynamic marker for miR‐21 degradation [20].

We next validated the cellular internalization and intracellular activation mechanism of Nb‐RIBOTAC using confocal microscopy in the selected PDAC cell lines. AF647‐labeled Nb‐RIBOTAC efficiently bound to EGFR on the cell surface and was internalized into the endolysosomal pathway within two hours of incubation. The efficient tumor‐targeting capability and favorable pharmacokinetics of the conjugate were further confirmed at the whole‐organism level. Non‐invasive in vivo imaging in PDAC xenograft models revealed rapid and selective tumor accumulation of AF647‐labeled Nb ‐RIBOTAC, with a strong signal detectable at tumor sites as early as 3 h post‐injection. The tumor‐to‐background ratio continued to increase, peaking at 24 h and remaining substantially elevated even at 48 h. This rapid targeting followed by a prolonged retention profile provided the pharmacokinetic basis for our dosing regimen, which was designed to maintain continuous target suppression by administering subsequent doses at 48‐h intervals.

The catalytic efficiency of the RIBOTAC platform proved superior to conventional antisense oligonucleotides, achieving >60% reduction of miR‐21 at low nanomolar exposure. The combination of rapid and precise tumor accumulation and conditional activation ensures that this high potency is confined to the target tissue, minimizing on‐target, off‐tumor effects and thereby enhancing the therapeutic window. Notably, this rationally designed dosing strategy translated into significant in vivo efficacy. Treatment with Nb‐RIBOTAC administered every 48 h led to profound suppression of tumor growth in multiple PDAC mouse models. This antitumor effect was correlated with a concomitant reactivation of tumor suppressor PDCD4. Importantly, these antitumor effects were achieved without observable toxicity in normal tissues, underscoring the safety advantage conferred by the enzyme‐selective activation strategy and the optimized dosing schedule.

## 4 Conclusion

In conclusion, we report herein the first proof‐of‐concept study of an RNA degradation approach to deplete oncogenic miR‐21 and disrupt PDAC progression in a tumor‐selective manner. The EGFR‐targeting Nb‐RIBOTAC as designed, Nb‐Fc‐Val‐Cit‐RIBOTAC, effectively degrades miR‐21 into EGFR‐high PDAC models to suppress tumor growth and restore tumor suppressor pathways. Notably, the catalytic efficiency of our Nb‐RIBOTAC demonstrated >60% miR‐21 knockdown at lower nanomolar concentrations than RIBOTAC alone, mainly due to the enhanced hydrophilicity and affinity. Furthermore, the tumor microenvironment‐responsive activation of the conjugate (via Val‐Cit linker cleavage) enabled cancer cell‐selective RNA degradation, achieving significant tumor suppression in vivo while sparing normal tissues.

We believe that the implications of this work extend beyond miR‐21 targeting. The modular strategy of antibody‐guided in vivo delivery of catalytic RNA degraders, combined with enzyme‐activatable payload release, can be adapted to target a wide range of oncogenic non‐coding RNAs, including microRNAs, lncRNAs, and oncofetal RNAs, which have elevational therapeutic targeting. By exchanging the nanobody domain, modifying the cleavable linker, or reprogramming the RNA‐recruiting moiety, this system can be adapted to various tumor antigens and RNA targets, offering a generalizable solution for “undruggable” oncogenic drivers in aggressive cancers. The novel design principles established here—combining nanobody targeting, conditional activation, and RNase recruitment—may be broadly adapted to other challenging RNA targets in oncology and beyond.

## Author Contributions

T.L. and X.W. conceived the idea for this work. Y.W. and T.L. discussed and conducted bioinformatics analysis. S.M., T.W., J.G.S. and R.B. discussed the research approach and strategy. T.L., D. C., Y. Z., Y., H. Y., T. J., H. J., M. Z., X. W., Z. W. and T.W. discussed the research approach and strategy. T.L. performed the in vitro experiments and analysed the data. T.L. and D. C. conducted the in vivo studies. F. G. and A. G. provided the CRC data. All authors discussed the results and commented on the paper.

## Funding

This research was supported by Shenzhen Medical Research Funds (C2303002, X.W.); startup grant (4937084, X.W.), direct grant (2024.175, X.W.), Faculty Postdoctoral Fellowship Scheme (FPFS/24‐25/053, FPFS/23‐24/061C, FPFS/23‐24/060, X.W.), and Research Committee – Group Research Scheme 2022–23 (WW/rc/grs2223/0560/23en, X.W.), by the Chinese University of Hong Kong; the Research Grants Council (AoE/M‐401/20, R4007‐23, C4024‐22GF, 14104223, 11103921, and 14111522, X.W.); Health and Medical Research Fund (08192166, X.W.); Jiangxi Overseas High‐Level Talent Project (20232BCJ25029, X.W.); grants by The Innovation and Technology Commission (PRP/049/22FX, C21200.260902008, H.T.M) and funding support by Research Talent Hub (PiH/478/23, T.L.).

## Ethics Statement

For the PDAC cohort, written informed consent was obtained from all the subjects, and the study was approved by the institutional review boards of Samsung Medical Center, and Nagoya University Graduate School of Medicine. The study protocol for our in‐house CRC cohort was reviewed and approved by the Ethics Committee Review Board of the Sixth Affiliated Hospital of Sun Yat‐sen University (Approval No. 2026ZSLYEC‐302; Project No. E2026152), in accordance with ICH–GCP principles and applicable Chinese regulations and guidelines. For ESCC, is available through the Genome Sequence Archive (GSA) in the BIG Data Center (http://bigd.big.ac.cn/gsa), Beijing Institute of Genomics (BIG), Chinese Academy of Sciences: HRA003107 (WGS & RNA‐seq, https://ngdc.cncb.ac.cn/gsa‐human/browse/HRA003107). All animal care and experimental procedures conducted in this study were approved by the Institutional Animal Care and Use Committee of Zunyi Medical University (Appl.No.: ZMU21‐2507‐002).

## Conflicts of Interest

The authors declare no conflicts of interest.

## Supporting information




**Supporting File 1**: advs76575‐sup‐0001‐SuppMat.pdf.

**Supporting File 2**: advs76575‐sup‐0002‐TableS1.xlsx.

**Supporting File 3**: advs76575‐sup‐0003‐TableS2.xlsx.

**Supporting File 4**: advs76575‐sup‐0004‐TableS3.xlsx.

**Supporting File 5**: advs76575‐sup‐0005‐TableS4.xlsx.

**Supporting File 6**: advs76575‐sup‐0006‐TableS5.xlsx.

**Supporting File 7**: advs76575‐sup‐0007‐TableS6.xlsx.

**Supporting File 8**: advs76575‐sup‐0008‐TableS7.xlsx.

**Supporting File 9**: advs76575‐sup‐0009‐TableS8.xlsx.

## Data Availability

The data that supports the findings of this study are available from the corresponding author upon reasonable request.

## ENDNOTES

1 Biomineralization of Metal‐Organic Framework Nanoparticle for Precise Delivery of Therapeutic PRoteins to Cancer Cells

2 Quadruplex mRNAs Silencing with Inducible Ribonuclease Targeting Chimera for Precision Tumor Therapy
