# Supplementary material for: Cell‐Selective Delivery of RIBOTACs via an Anti‐EGFR Nanobody for Pancreatic Cancer Treatment
Source: Adv Sci (Weinh). 2026 Jul 16;13(55):e76575. doi: 10.1002/advs.76575 (PMC13373894; doi:10.1002/advs.76575)
Supplement: Supplementary file 1 [file ADVS-13-e76575-s009.pdf]

Supplementary information

Cell-Selective Delivery of RIBOTACs via an anti-EGFR Nanobody for Pancreatic Cancer Treatment

General

All reagents for chemical synthesis were purchased from commercial sources and used as received. All cell lines used in this study were purchased from Cell Resource Center, Peking Union Medical College (Beijing, China). The cells were cultured in DMEM supplemented with 10% (v/v) fetal bovine serum (FBS) and 1% penicillin/streptomycin at 37 °C in the presence of 5% CO_2. The cells were regularly sub-cultured with trypsin-EDTA (0.25%, w/w), the cell density was determined using hemocytometer. For protein degradation and intracellular delivery study, the cells were seeded in 6-well, 48-well or 96-well plates 24 h prior to experiments.

Materials

All commercially available chemicals were purchased from Sigma-Aldrich (St. Louis, MO), Innochem (Beijing, China) and Aladdin (Beijing, China). Kanamycin, ampicillin, isopropyl-β-D-thiogalactopyranoside (IPTG) and phenylmethylsulphonyl fluoride (PMSF) were purchased from Solarbio (Beijing, China). LysoTracker Green was purchased from Beyotime (Shanghai, China). Bovine serum albumin (BSA) was purchased from Sigma-Aldrich (St. Louis, MO). Antibodies used in this study were anti-EGFR (HY-P80116, MCE), anti-PDCD4(HY-P80269, MCE), GAPDH (ab8245, abcam). Antibody, anti-rabbit/mouse IgG HRP-linked antibodies (7074S/7076S, CST, USA). Cell Counting Kit-8 was purchased from Beyotime (Shanghai, China).
Western blot images were taken on a Tanon 5200 Multi Imaging System. Confocal laser scanning microscopy images were obtained from OLYMPUS FV1000-IX81. Other equipment: thermomixer (Thermomixer, MS-100), centrifuge (Eppendorf, 5430R), NanodropONE (Thermo), Zeba desalting columns (Thermo, 89890/89892/89883), LC-MS (Waters BioAccord), LC-MS column (ACQUITY UPLC Protein BEH C4, 300 Å, 1.7 µm, 2.1 × 50 mm).

Cell culture

PANC-1, ASPC-1, S2VP10, PK-8, MIA PaCa-2, KLM and normal cell HPNE and MCF-10A cells were purchased from National Infrastructure of Cell Line Resource (Beijing, China). PANC-1, ASPC-1, S2VP10, PK-8, MIA PaCa-2, KLM and normal HPNE cells were maintained in DMEM (Dulbecco's Modified Eagle's Medium) supplemented with 10% FBS (fetal bovine serum) and 1% penicillin/streptomycin. MCF-10A cells were cultured in MCF-10A Cell Complete Medium.

Small RNA sequencing, candidate miRNA discovery, and validation

For the in-house cohorts of CRC, ESCC, and PDAC, small RNA sequencing data were processed using the Cutadapt pipeline (v2.2) to remove adapter sequences and filter out low-quality reads. Cleaned reads were subsequently aligned to reference miRNA sequences from miRBase[1], and miRNA expression was quantified using the miRDeep2[2]. For STAD, publicly available small RNA sequencing data was retrieved from The Cancer Genome Atlas (TCGA) database using the TCGAbiolinks R package. For all cohorts, miRNA abundance was normalized and expressed as counts per million (CPM). Differential expression analysis between tumor and matched adjacent normal tissues was performed using the R package limma.

Following individual cancer-type analyses, we further identified commonly dysregulated miRNAs across the four gastrointestinal cancers by intersecting significantly upregulated candidates. To validate the expression patterns of candidate miRNAs, additional independent public datasets were downloaded from the Gene Expression Omnibus (GEO) database, including three microarray datasets (GSE115513, GSE13937, and GSE23739) and one small RNA-seq dataset (GSE119794). For GSE13937, only ESCC samples were included in the analysis. Because these validation datasets were generated using different technologies, preprocessing was performed in a dataset-specific manner. For microarray datasets, sample annotations were manually curated to define tumor and normal groups, and expression matrices were cleaned and normalized before downstream analysis. For the small RNA-seq validation dataset, count data were processed using a standard RNA-seq workflow, including low-count filtering, TMM normalization, and voom transformation. In the validation cohorts, expression differences of miR-21-3p and miR-21-5p between paired tumor and normal samples were assessed using paired Wilcoxon signed-rank tests.

Statistical analysis

Statistical analyses were conducted using R software (version 4.2.1, https://cran.r-project.org/). Pearson correlation coefficients and corresponding p values were calculated to assess the relationship between miR-21-5p and miR-21-3p expression across the four cancers. The Pearson correlation coefficient was computed to assess the linear relationship between variables miR-21-3p and miR-21-5p. Statistical significance was evaluated using a two-tailed t-test under the null hypothesis that the population correlation is zero. For survival analyses, patients were stratified into high- and low-expression groups based on the optimal cutoff value determined by the survminer R package. Kaplan–Meier survival curves were generated, and statistical significance was assessed using the log-rank test. A p-value < 0.05 was considered statistically significant unless otherwise specified.

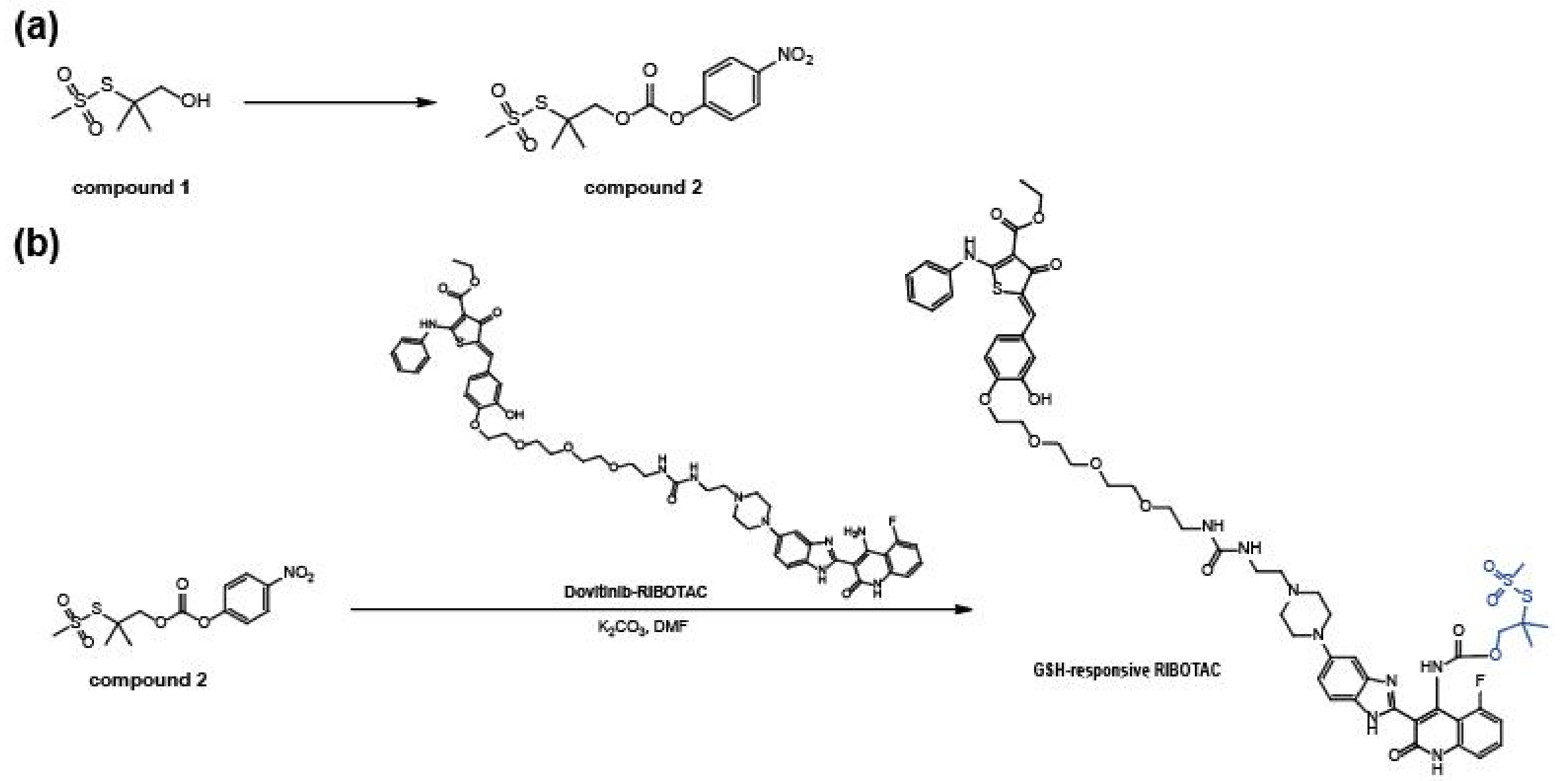

Scheme S1. Synthesis route of GSH-responsive RIBOTAC.

Synthesis of compound 2

To the mixture of compound 1 (20 mg, 108.53 µmol, 1 eq) in DCM (0.3 mL) was added pyridine (25.75 mg, 325.60 µmol, 26.28 µL, 3 eq) and (4-nitrophenyl) carbonochloridate (43.75 mg, 217.07 µmol, 2 eq). After addition, the mixture was stirred at 20°C for 1 h. TLC showed a new spot (Rf=0.19, hexane: ethyl acetate=4:1). The mixture was purified directly by pre-TLC (hexane: ethyl acetate=4:1) to give compound 2 (23 mg) as colorless gum. ^{1}H NMR (400 MHz, $CDCl_3$) δ= 8.31 (d, J = 9.1 Hz, 2H), 7.42 (d, J = 9.1 Hz, 2H), 4.57 (s, 2H), 3.42 (s, 3H), 1.66 (s, 6H)

Synthesis of GSH-responsive RIBOTAC

To the mixture of Dovitinib-RIBOTAC (5 mg, 4.97 µmol, 1 eq) and compound 2 (2.60 mg, 7.45 µmol, 1.5 eq) in DMF (0.5 mL) was added K_2CO_3 (2.06 mg, 14.91 µmol, 3 eq) under ice bath. After addition, the mixture was stirred at 20°C for 20 min. The mixture was filtered; the filtrate was purified by pre-HPLC to give GSH-responsive RIBOTAC (1.7 mg) as yellow solid. Pre-HPLC method:column: Phenomenex Luna C18 100×30mm×5µm; mobile phase: [H_2O (0.1% TFA)-ACN]; gradient: 35%-55% B over 8.0 min. 1H NMR (400 MHz, DMSO-d_6) δ = 12.82 (br d, *J* = 4.3 Hz, 1H), 11.61 (s, 1H), 11.29 (s, 1H), 10.49 (s, 1H), 8.80 (d, *J* = 3.3 Hz, 1H), 7.60 - 7.41 (m, 14H), 7.39 - 7.23 (m, 4H), 7.19 (br d, *J* = 8.3 Hz, 1H), 7.12 - 6.96 (m, 3H), 6.91 - 6.77 (m, 3H), 6.34 - 6.23 (m, 2H), 5.31 (s, 2H), 4.43 (s, 2H), 4.34 - 4.23 (m, 2H), 4.21 - 4.13 (m, 2H), 3.83 - 3.62 (m, 6H), 3.58 - 3.50 (m, 10H), 3.06 - 2.93 (m, 3H), 2.17 (s, 4H), 1.52 (s, 6H), 1.29 (t, *J* = 7.1 Hz, 3H).

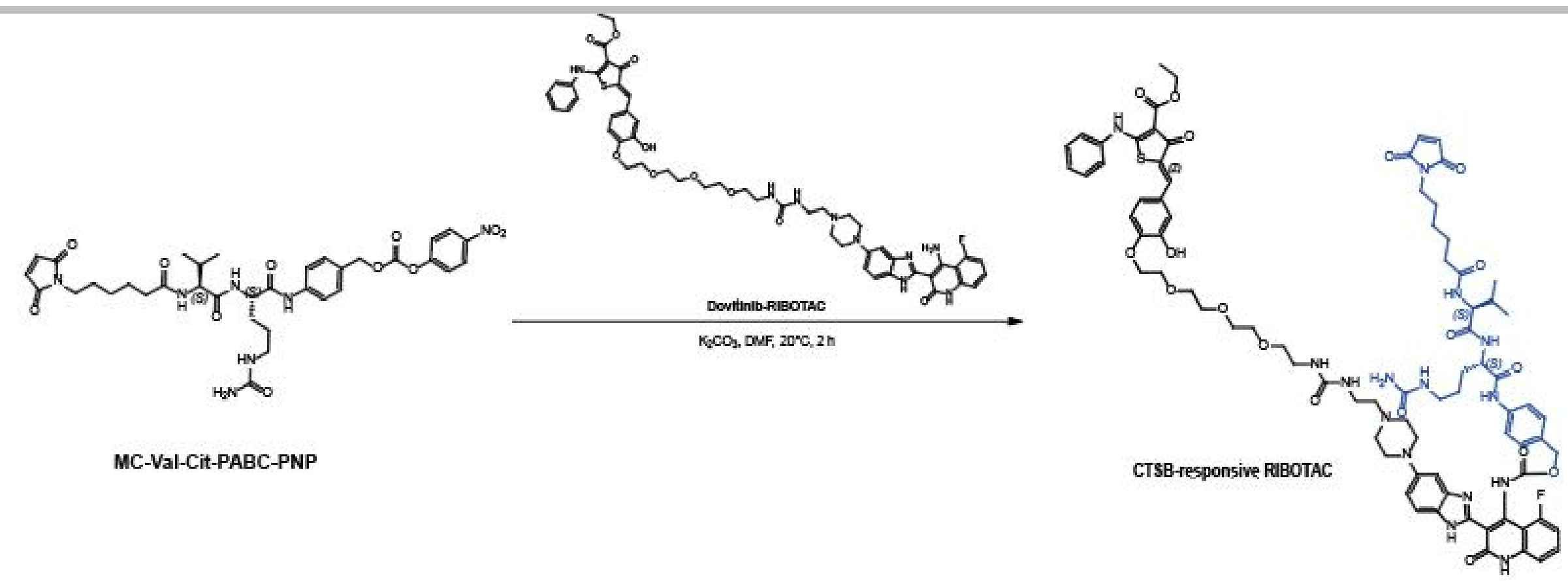

Scheme S2. Synthesis route of CTSB-responsive RIBOTAC.

Synthesis of CTSB-responsive RIBOTAC

To the mixture of Dovitinib-RIBOTAC (17 mg, 16.90 μmol, 1 eq) and 159857-81-5 (18.70 mg, 25.35 μmol, 1.5 eq) in DMF (1 mL) was added K_2CO_3 (4.67 mg, 33.79 μmol, 2 eq) under ice bath. After addition, the mixture was stirred at 20°C for 2 h. The mixture was filtered, the filtrate was purified by pre-HPLC to give CTSB-responsive RIBOTAC (3.5 mg) as yellow solid.^{1}H NMR (400 MHz, DMSO-d6) δ12.82 (br d, J = 0.9 Hz, 1H), 11.61 (s, 1H), 11.28 (s, 1H), 10.06 (s, 1H), 9.73 - 9.53 (m, 1H), 8.14 - 8.07 (m, 1H), 7.86 - 7.71 (m, 2H), 7.69 - 7.49 (m, 8H), 7.47 - 7.31 (m, 5H), 7.29 (s, 1H), 7.24 - 7.18 (m, 3H), 7.15 - 7.02 (m, 3H), 6.98 (d, J = 11.8 Hz, 5H), 6.38 - 6.23 (m, 2H), 6.04 - 5.95 (m, 1H), 5.43 (br s, 2H), 5.18 (s, 2H), 4.45 - 4.36 (m, 1H), 4.33 - 4.24 (m, 2H), 4.20 (br t, J = 7.8 Hz, 1H), 4.14 (br s, 2H), 3.84 - 3.59 (m, 6H), 3.55 - 3.47 (m, 8H), 3.25 - 3.12 (m, 6H), 3.07 - 2.92 (m, 4H), 2.15 (br d, J = 15.5 Hz, 3H), 2.01 - 1.93 (m, 1H), 1.73 - 1.66 (m, 1H), 1.65 - 1.58 (m, 1H), 1.54 - 1.36 (m, 6H), 1.30 (t, J = 7.1 Hz, 3H), 1.25 - 1.12 (m, 3H), 0.84 (br dd, J = 6.5, 12.6 Hz, 6H). 1F NMR (376.5 MHz, DMSO-d6) δ-73.519, -114.10. Pre-HPLC method: column: Phenomenex Luna C18 100×30mm×5μm; mobile phase: [H_2O (0.1% TFA)-ACN]; gradient: 30%-50% B over 10.0 min. ^{1}F NMR (376.5 MHz, DMSO-d_6) δ-73.519, -114.10.

Construction of RIBOTAC with VHH-hIgG1 Fc nanobody

Antibody JOT0009-1-Fc (10.2 mg, 3 mg/mL in 40 mM PB, pH 7.0) was treated with TCEP (5 mM, 0.078 mL) at 25°C for 16 hours. CTSB-responsive RIBOTAC (10 mg/mL in DMSO, 0.146 mL) was added, and the mixture was incubated at 22°C for 3 hours. The reaction was quenched with cysteine (50 mM, 0.069 mL) at 22°C for 20 minutes. The solution was buffer exchanged into 40 mM PB (pH 7.0) via ultrafiltration (yield: 49%; final purity: 92.11%).

Surface plasmon resonance (SPR) analysis

SPR analysis was performed using a Biacore 8k (Cytiva) instrument at 25 °C. A CM5 sensor chip was active by using sulpho-N-hydroxysuccinimide (NHS)/1-ethyl3-(3-dimethylaminopropyl) carbodiimide (EDC) chemistry. The chip was immobilized with the recombinant EGFR with a concentration of 5 μg/mL in sodium acetate, pH 4.5. Various concentrations of VHH-FC (or Nb-RIBOTAC) were injected at a flow rate of 30 μL/min in PBS-T (137 mM NaCl, 2.7 mM KCl, 10 mM Na_2HPO_4, 1.8 mM KH_2PO_4, and 0.005% (v/v) Surfactant P20) running buffer. The results were analyzed with the BiacoreTM Insight Evaluation software (version 3.0.12). The binding affinity (KD) was calculated as KD (nM) = Kd (1/s) / Ka (1/Ms), where Kd is the dissociation constant and Ka is the association constant.

The half-maximal effective concentration (EC_{50}) experimental procedures

Antigen Coating: Dilute the EGFR antigen to 1 µg/mL with PBS, add 100 µL of the diluted antigen to each well, and incubate overnight at 4 °C for 16 hours.
Discard the coating solution, wash the microplate 3 times with PBST using a microplate washer, and pat dry the residual liquid in the wells.
Blocking: Add 200 µL of 4% non-fat dry milk to each well, incubate at room temperature for 2 hours, then wash the plate 3 times and pat dry.
Serial Dilution and Incubation of Antibodies：

a. Perform gradient dilution of VHH-FC (or Nb-RIBOTAC) (stored at -20°C) with PBS to obtain the concentration gradient: 2048, 1024, 512, 128, 32, 8, 2, 0 ng/mL (8 concentration points in total).
b. Add 100 µL of the diluted antibody sample to each well, and add 100 µL of PBS to the blank control wells. Incubate at room temperature for 2 hours.
c. Wash the microplate 5 times and pat dry.

HRP-conjugated Secondary Antibody Incubation: Dilute the HRP-conjugated secondary antibody 1:3000 with PBS, add 100 µL to each well, and incubate at room temperature for 1.5 hours. Wash the plate 5 times and pat dry.
Add 100 µL of TMB chromogenic solution to each well, and incubate in the dark at room temperature for 1.5 minutes. Add 100 µL of stop solution to each well to terminate the color reaction and read the absorbance at OD450nm using a microplate reader immediately.

CTSB enzymatic cleavage in solution

Determination of the temporal interval needed for the enzymatic cleavage of CTSB was executed as follows:
Recombinant cathepsin B (CTSB) was activated in 20 mM MES buffer (pH 5.0) containing 1 mM TCEP at 37 °C for 15 min, then mixed with the Nb-RIBOTAC (1 mg/ml) at an enzyme-to-substrate molar ratio of 1:5 (CTSB final concentration: 2.5 µM). The contents of the vial were vigorously mixed and then incubated at 37 °C for a series of defined time points,0，3，6，8. The CTSB inhibitor CA-074 methyl ester is added as a negative control at 0h and detected at 8 h. At each time point, aliquots were quenched with three volumes of ice-cold acetonitrile containing 0.1% formic acid, centrifuged, and analyzed by PLRP-HPLC, UV detection at 280 nm. The integrity of Nb-RIBOTAC was assessed by monitoring the decrease of the intact Nb-RIBOTAC peak area, while the appearance of peaks corresponding to released VHH-Fc was quantified relative to total peak area.

AF647 antibody labeling

The remaining antibody required for labeling was transferred into a centrifuge tube. Based on calculated amounts, appropriate volumes of AF647 and antibody modifier were added to the antibody solution, mixed thoroughly by repeated pipetting or vortexing, and incubated at room temperature for 2 hours. After incubation, the reaction mixture was transferred into an ultrafiltration tube and centrifuged to remove unbound free components. The purified conjugate was then collected into an EP tube, supplemented with preservative if necessary, and stored at 4°C protected from light.

Western blot analysis.

Cells were seeded in 6-well plates at a density of 2×10^5 cells per well, one day prior to the experiment. On the day of the experiment, the cells were treated with Nb-RIBOTAC at a final concentration of 500

nM. Following a 12-hour incubation period, the cells were lysed using a buffer consisting of 50 mM Tris, 150 mM NaCl, 1% NP-40, 0.25% sodium deoxycholate, and 1 mM PMSF at pH 7.4. The lysate was then used for a western blot assay to study the abundance of proteins.

Downregulation and quantitative real-time PCR (RT-qPCR) of miR-21

Cells were separately cultured in six-well plates for 24 h followed by the addition of RIBOTAC or Nb-RIBOTAC for 48 h incubation. The RNA was then extracted from the cells with SanPrep Column microRNA Extraction Kit and amplified by miRNA First Strand cDNA Synthesis (Tailing Reaction) for quantification with MicroRNAs qPCR Kit (SYBR Green Method). The amplified RNA miR-21 was also quantified by agarose gel electrophoresis. All procedures are strictly in accordance with the kit instructions.

CCK-8 cell proliferation and cytotoxicity assay

Cells were plated in 96-well plates at a density of 1.0×10^4 cells per well, 24 hours prior to the delivery experiment. Subsequently, the cells were incubated with Nb-RIBOTAC at varying concentrations, as indicated, for a duration of 48 hours at 37 °C. Following the incubation period, 10 μL of CCK-8 solution was added to each well. The cells were then cultured for an additional 1 hour at 37 °C, after which the absorbance was measured at 450 nm using a microplate reader (BioTek Synergy H1, USA) to determine cell viability.

Intracellular distribution study of Nb-RIBOTAC

Cells were seeded at a density of 8×10^4 cells per mL in a glass bottom cell culture dish 24 hours prior to the experiment. The cells were then incubated with Nb-RIBOTAC at different time points at 37 °C. After the incubation, the cells were washed twice with a 3 mg/mL solution of heparin sodium. Subsequently, the cells were incubated with 25 nM Lyso-TrackerGreen for 15 minutes at 37 °C. Following this, the cells underwent two additional washes with DPBS before being imaged using confocal laser scanning microscopy (CLSM). Second-Generation Transcriptome Library Construction and Sequencing - BGI

RNA-seq study

Denatured Total RNA is thermally denatured to disrupt secondary structures, and mRNA is enriched using oligo(dT) magnetic beads. Fragmentation reagent is added to the mRNA obtained in the previous step, and the mixture is incubated at a suitable temperature for a defined duration to fragment the mRNA. cDNA Synthesis: A pre-prepared first-strand synthesis reaction mix is added to the fragmented mRNA. The first strand of cDNA is synthesized in a PCR instrument according to a specific program. Subsequently, a second-strand synthesis reaction mix is prepared, and the reaction is incubated at a suitable temperature for a defined duration to synthesize the second strand of cDNA. A-Tailing, and Adapter Ligation A reaction mix are prepared to repair the ends of the double-stranded cDNA, which is incubated at a suitable temperature for a defined duration. An "A" base is then added to the 3' ends. Next, an adapter ligation reaction mix is prepared and incubated at a suitable temperature for a defined duration to ligate adapters to the cDNA. PCR Amplification: A PCR reaction mix is prepared, and the reaction program is set to amplify the ligation products. The PCR products are denatured into single strands. A circularization reaction mix is prepared, thoroughly mixed, and incubated at a suitable temperature for a defined duration to generate single-stranded circular products. After digesting any non-circularized linear DNA molecules, the final library is obtained. Library QC: The library's fragment size and concentration

are detected using the Agilent 2100 Bioanalyzer. The PCR products are denatured into single strands. A circularization reaction mix is prepared, thoroughly mixed, and incubated at a suitable temperature for a defined duration to generate single-stranded circular products. After digesting any non-circularized linear DNA molecules, the final library is obtained. DNB Generation and Sequencing Single-stranded circular DNA molecules are amplified via rolling circle replication to form DNA Nanoballs (DNBs), each containing over 200 copies. The resulting DNBs are loaded into the microwells of a high-density DNA nanochip using high-density DNA nanochip technology. Sequencing is performed using Combinatorial Probe-Anchor Polymerization (cPAS). Library Prep Kit: VAHTS Universal V6 RNA-seq Library Prep Kit for MGI® (#NRM605). Sequencing Platform: DNBSEQ-T7.

Sample size justification

A priori analysis was performed using G*Power (version 3.1.9.7) to estimate the number of animals required to detect differences in whole-body radiance between treatment groups at a given time point after inoculation. Assuming a two-tailed independent-samples *t*-test, a significance level of 0.05, and 80% power, the minimum required sample size was estimated to be 4 mice per group, based on an expected effect size of 2.8 derived from previously published studies using a similar model. Therefore, the sample size used in this study (n = 5 per group) exceeded the minimum estimated requirement.

***In vivo* delivery of Nb-RIBOTAC**

All animal care and experimental procedures conducted in this study were approved by the Institutional Animal Care and Use Committee of Zunyi Medical University (Appl.No.: ZMU21-2507-002). All subsequent procedures were carried out in compliance with relevant institutional and national animal welfare policies. To generate PANC-1 tumor-bearing mouse xenograft for studying the apoptosis induction effect *in vivo*, 4–6-week-old female Nu/Nu nude mouse (purchased from Beijing Vital River Laboratory Animal Technology Co., Ltd) was subcutaneously injected with PANC-1 cells (5×10^6 cells) suspended in 50 μL phosphate-buffered saline (PBS) and mixed with Corning® Matrigel® to the left axilla region. For the *in vivo* biodistribution study of Nb-RIBOTAC, PANC-1 tumor-bearing mice with tumor size of ~100 mm^3 were intravenously injected with 100 μL Nb-RIBOTAC at the same dose of 2.5 mg/kg at 3, 6, 24 and 48h. Mice were imaged and sacrificed 48 h post-injection, the organs including heart, liver, spleen, lung, kidney, and tumor were collected for fluorescence imaging on IVIS small animal imaging system (Perkin Elmer, USA).

For studying the *in vivo* anticancer efficacy of Nb-RIBOTAC, PANC-1 tumor-bearing mice with tumor size of ~100 mm^3 were randomly divided into four groups (5 mice in each group) and intravenously injected with PBS, Nb-RIBOTAC, Nb, RIBOTAC (all formulated in a solution composed of 0.1% Tween-20) once every two days. Tumor size and mouse body weight were measured every two days. The tumor volume was calculated using the following formula: $length\times width^2/2$ and normalized to that of mouse before injections. At the end of treatment, mouse blood was withdrawn to collect serum for liver toxicity and biocompatibility study of different injections. Meanwhile, tumor tissues were collected to measure PDCD4 change using western blot assay. At the end of the study, the mice were euthanized, and blood samples were collected and centrifuged to isolate serum for liver function assays. Additionally, mouse tissues, including the tumor, were harvested for further analysis, including western blot assays, TUNEL staining for apoptosis detection, and H&E staining for histological examination.

Table S1. Differentially expressed miRNAs between tumor and normal samples in colorectal cancer (CRC) cohort. (Full data available in Table S1.xlsx)

Table S2. Differentially expressed miRNAs between tumor and normal samples in esophageal squamous cell carcinoma (ESCC) cohort. (Full data available in Table S2.xlsx)

Table S3. Differentially expressed miRNAs between tumor and normal samples in pancreatic ductal adenocarcinoma (PDAC) cohort. (Full data available in Table S3.xlsx)

Table S4. Differentially expressed miRNAs between tumor and normal samples in stomach adenocarcinoma (STAD) cohort. (Full data available in Table S4.xlsx)

Table S5. Comparison of the property of two antibodies. (Full data available in Table S5.xlsx)

Table S6. Size-exclusion chromatography of Nb-RIBOTAC. (Full data available in Table S6.xlsx)

Table S7. Results of differential expression between Nb-RIBOTAC-treated and untreated PANC-1 cells. (Full data available in Table S7.xlsx)

Table S8. KEGG pathway enrichment data. (Full data available in Table S8.xlsx)

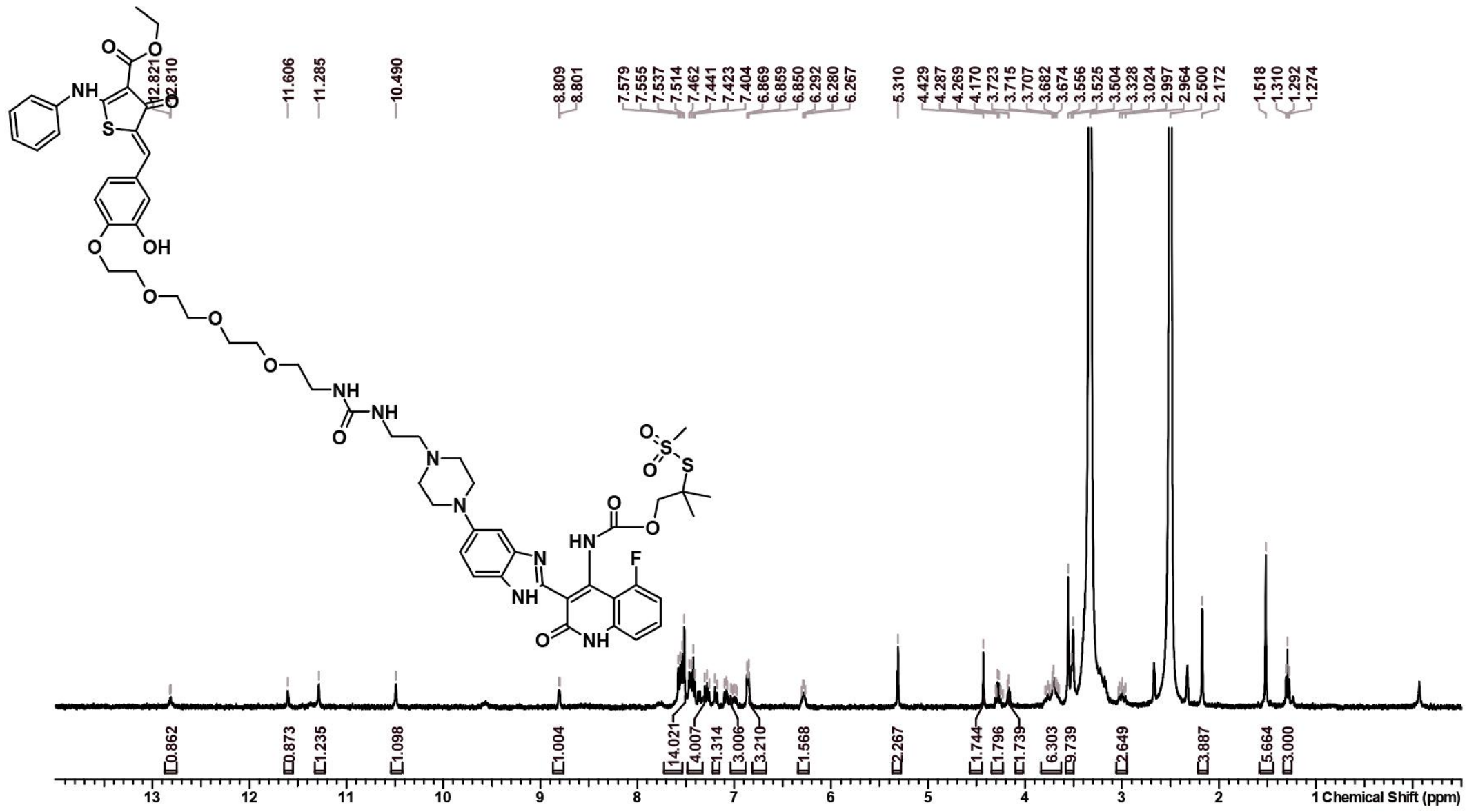

Figure S1. ^{1}H NMR spectrum (300 MHz, Chloroform-*d*, 298 K) of GSH-responsive RIBOTAC.

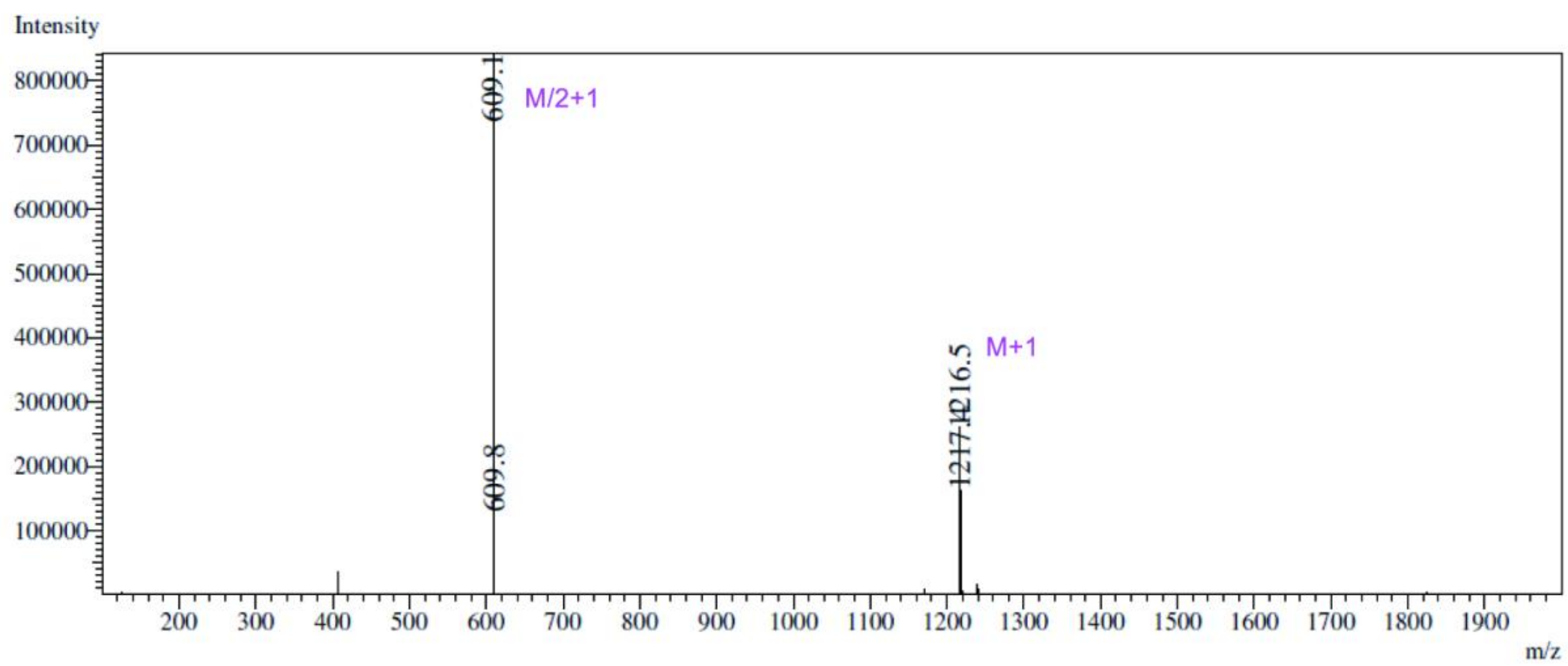

Figure S2. ESI-MS of GSH-responsive RIBOTAC.

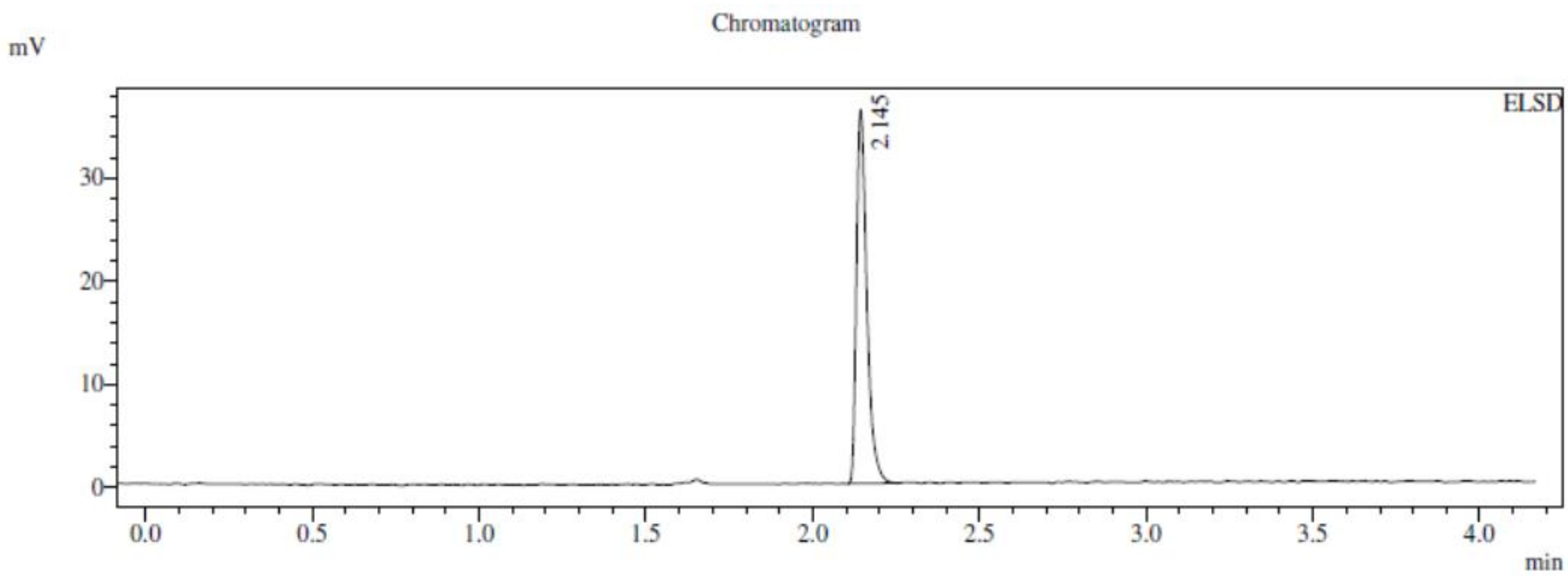

Figure S3. LCMS spectrum of GSH-responsive RIBOTAC

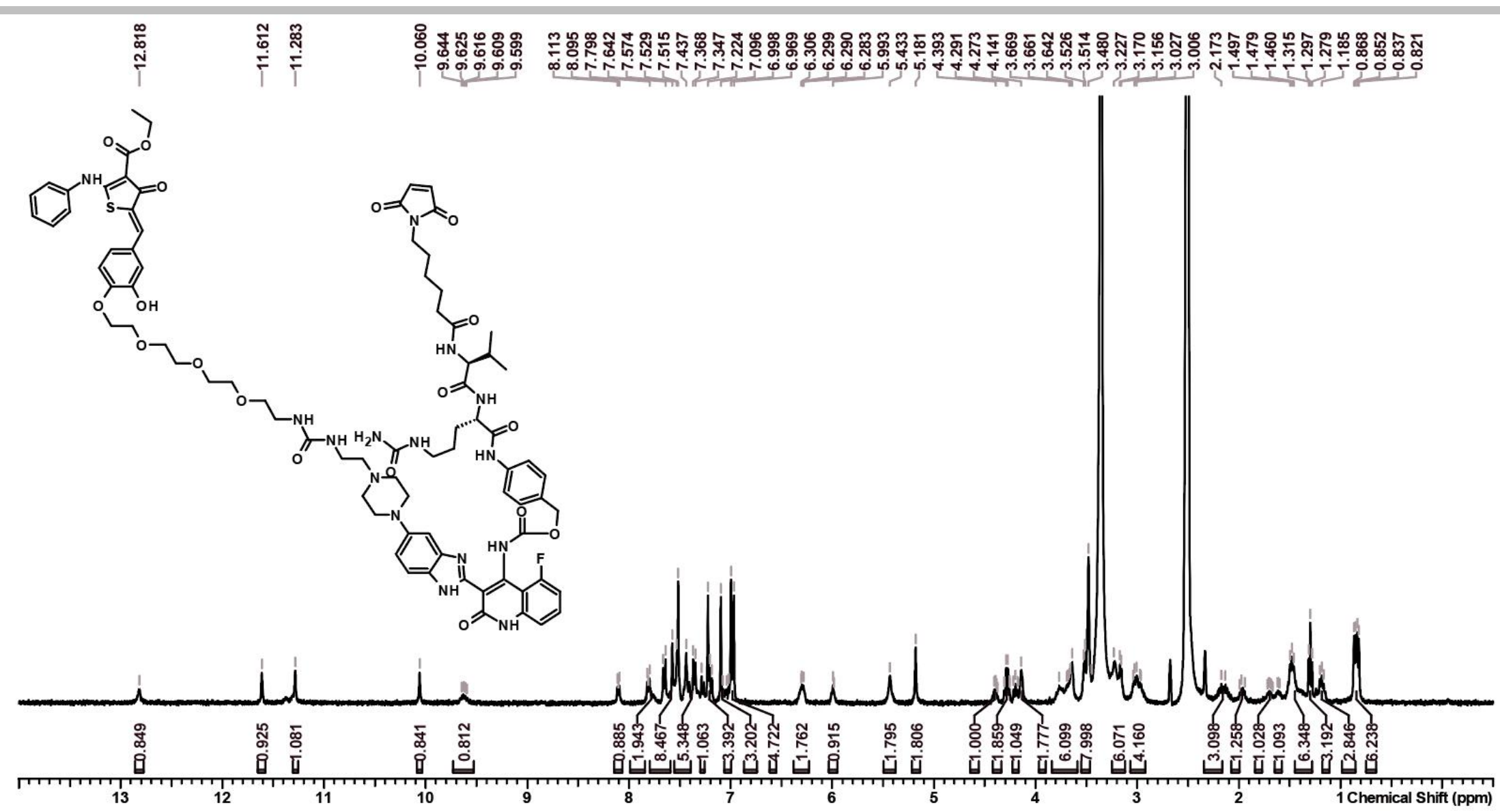

Figure S4. ^{1}H NMR spectrum (300 MHz, Chloroform-*d*, 298 K) of CTSB-responsive RIBOTAC.

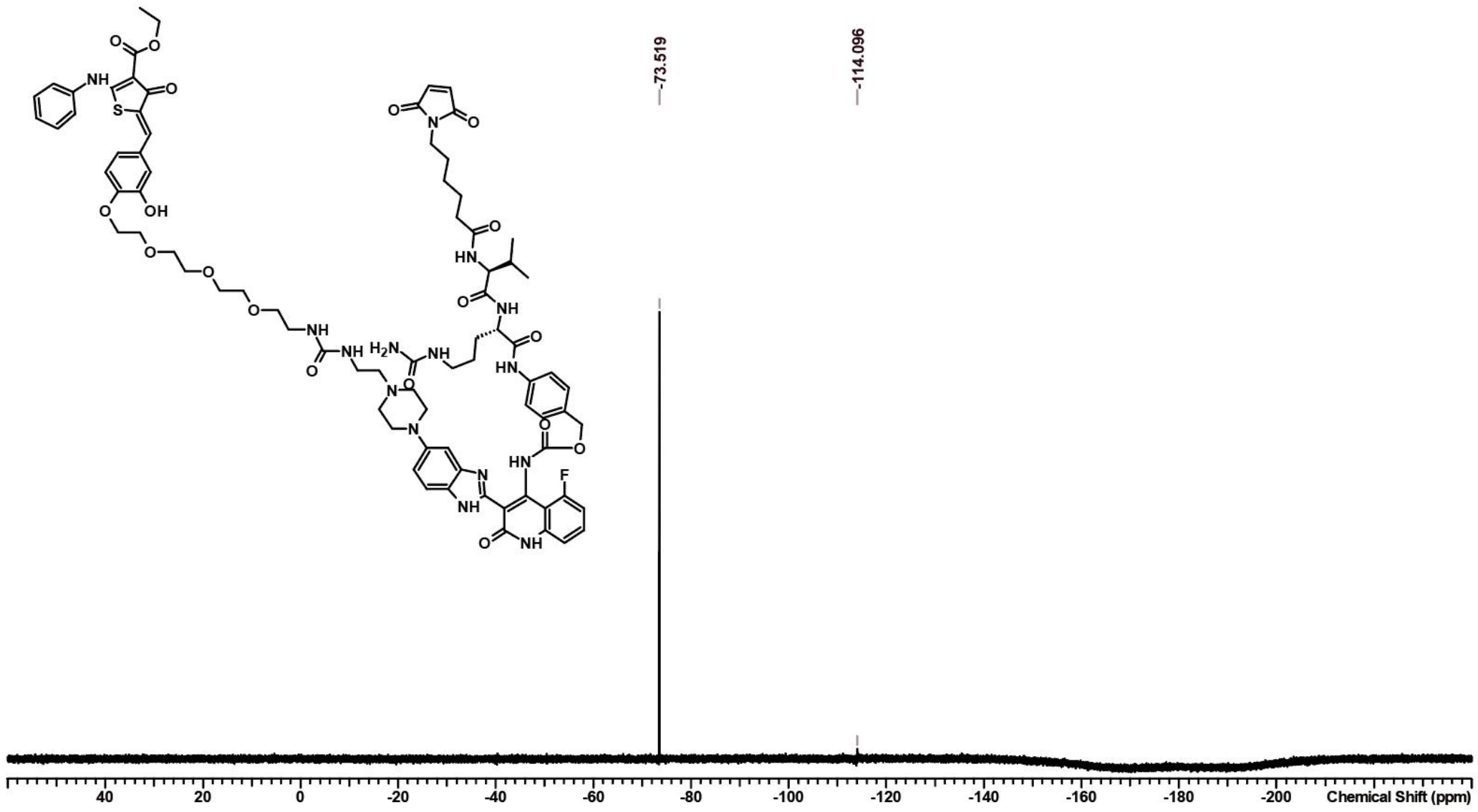

Figure S5. ^{19}F-NMR spectrum of CTSB-responsive RIBOTAC.

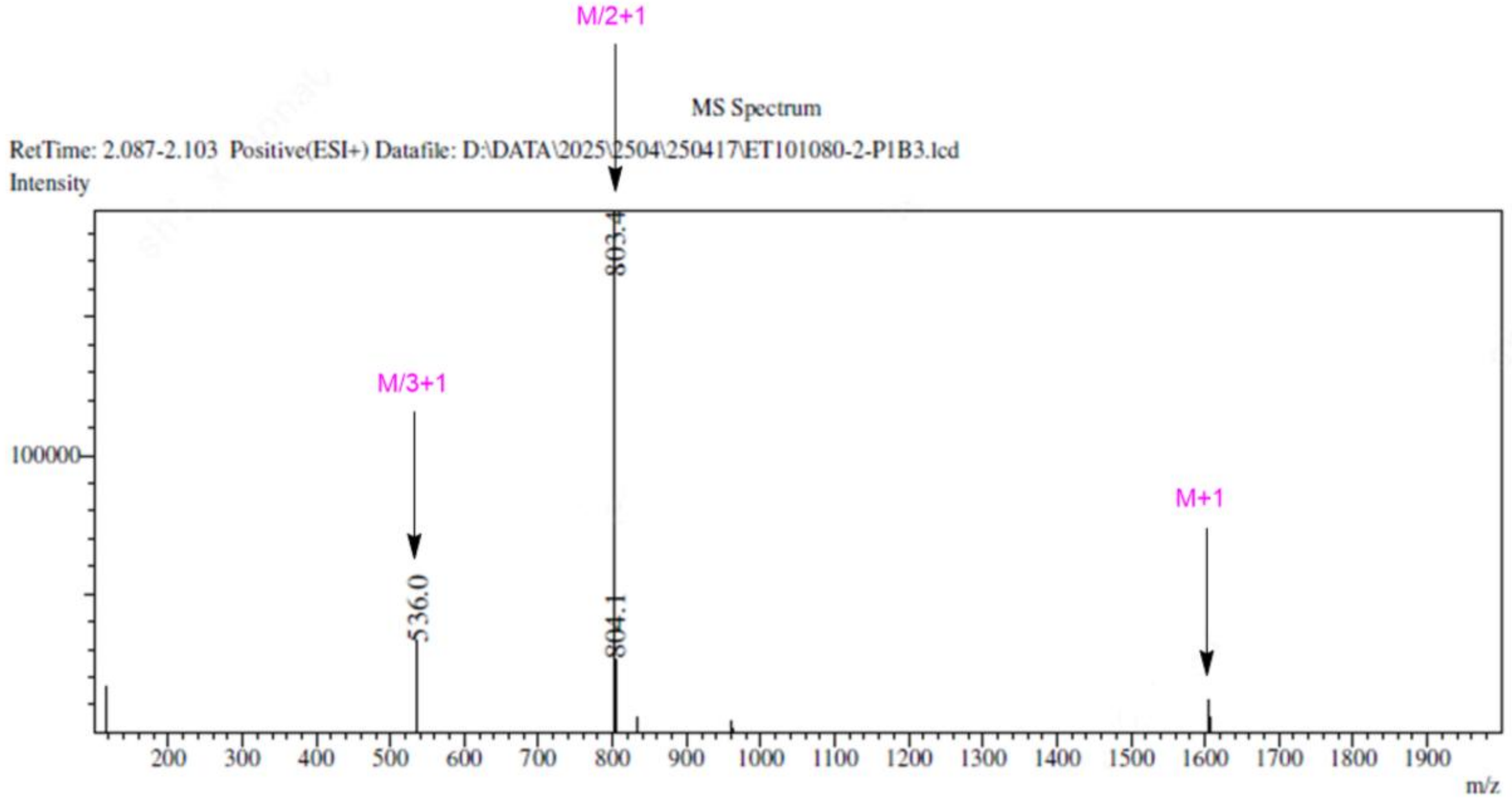

Figure S6. ESI-MS of GSH-responsive RIBOTAC.

.

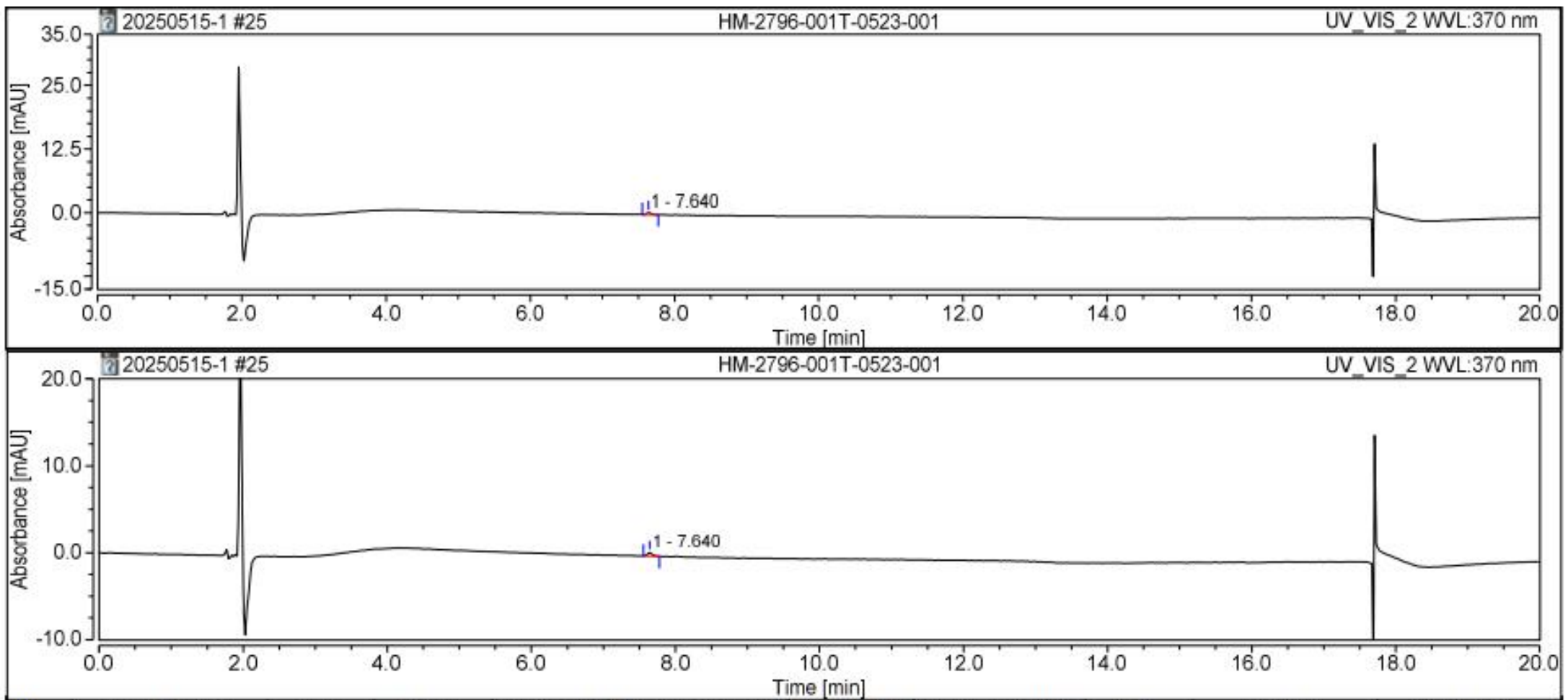

No.	Retention Time min	Area mAU*s	Height mAU	Peak Width min	Tailing factor(USP)	Plates (USP)	Resolution (USP)	S/N	Rel.Area %	Peak Name
1	7.640	2.027	0.444	0.11	1.37	77870	n.a.	n.a.	100.00	
Total:		2.027	0.444		1.37	77870.00				

Figure S7. Chromatography-Free-drug

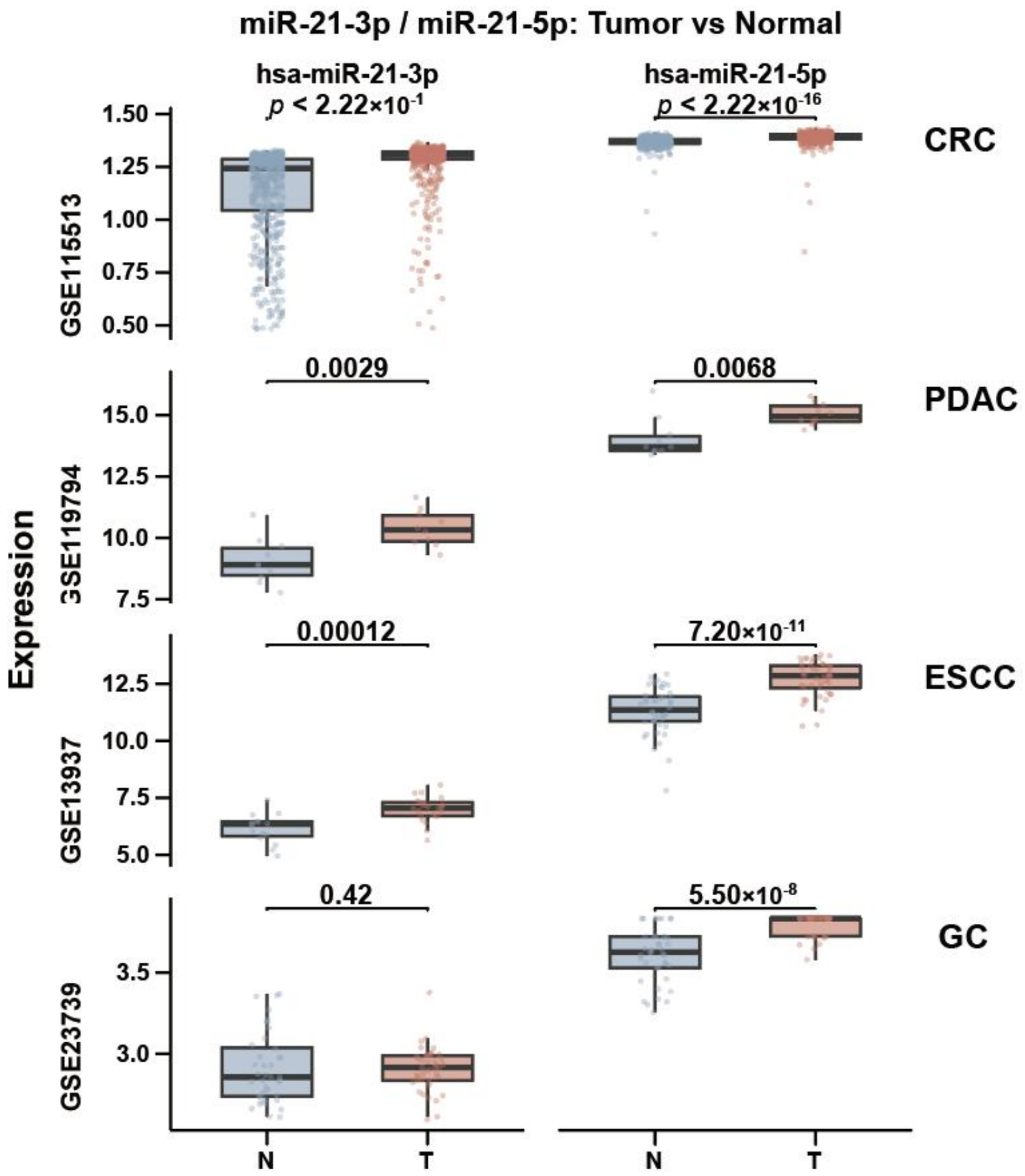

Figure S8. Validation across multiple independent public datasets demonstrated that both miR-21-3p and miR-21-5p were generally elevated in tumor tissues relative to adjacent normal controls in CRC, PDAC, ESCC, and GC, respectively.

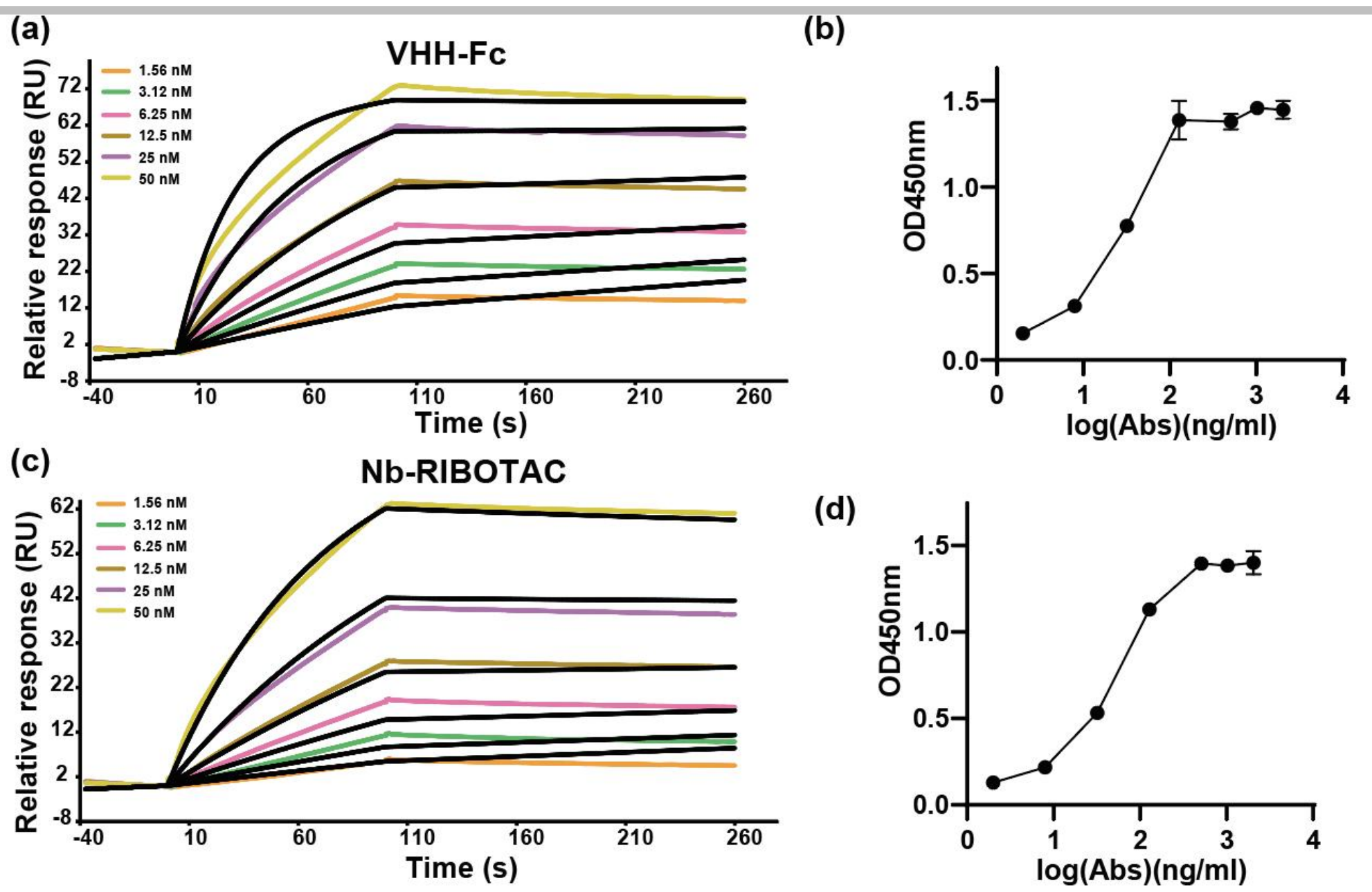

Group	Immobilized li-gand	Quality Kinetics Chi² (RU²)	1:1 binding Ka (1/Ms)	Kd (1/s)	KD (M)	Rmax (RU)	tc	EC_{50} (ng/mL)
VHH-Fc	5 ug/mL EGFR	6.37	7.79×10^{5}	9.07×10^{4}	1.16×10^{9}	66.5	3.71×10^{12}	29.33
NbRIBOTAC	5 ug/mL EGFR	3.06	2.81×10^{5}	6.40×10^{4}	2.28×10^{9}	81.6	9.50×10^{11}	54.38

Figure S9. Surface plasmon resonance (SPR) and half-maximal effective concentration (EC50) analysis of VHH-Fc and Nb-RIBOTAC. **(a)** SPR and (**b)** EC_{50} result of VHH-Fc. **(c)** SPR and (**d)** EC_{50} result of Nb-RIBOTAC.

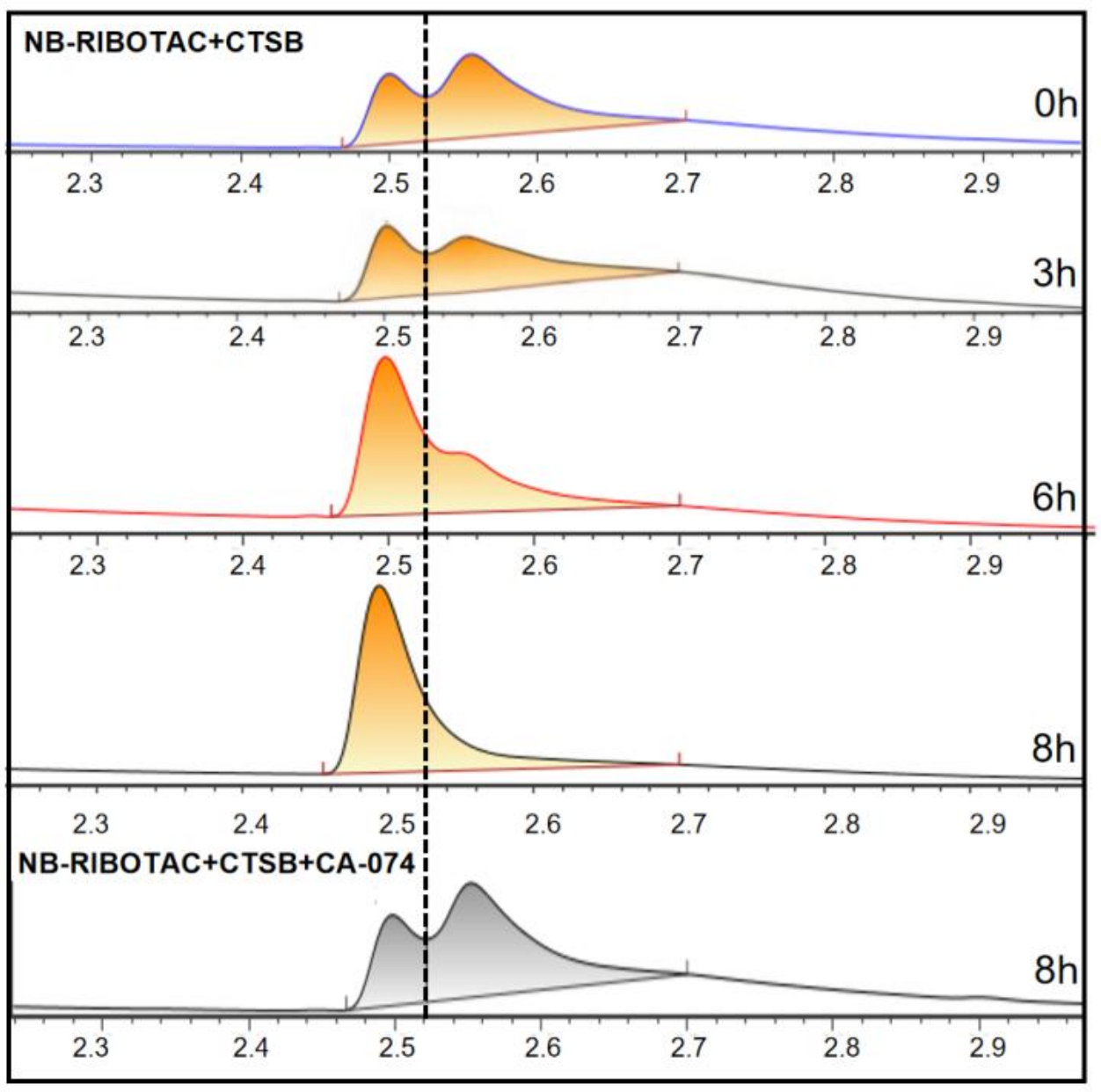

Figure S10. In vitro cathepsin B–mediated cleavage kinetics of the Val-Cit linker.

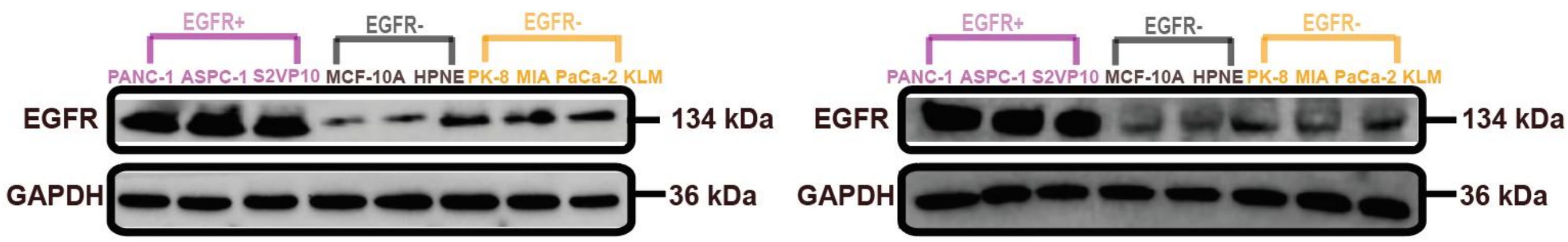

Figure S11. Full replicates of Figure 3b.

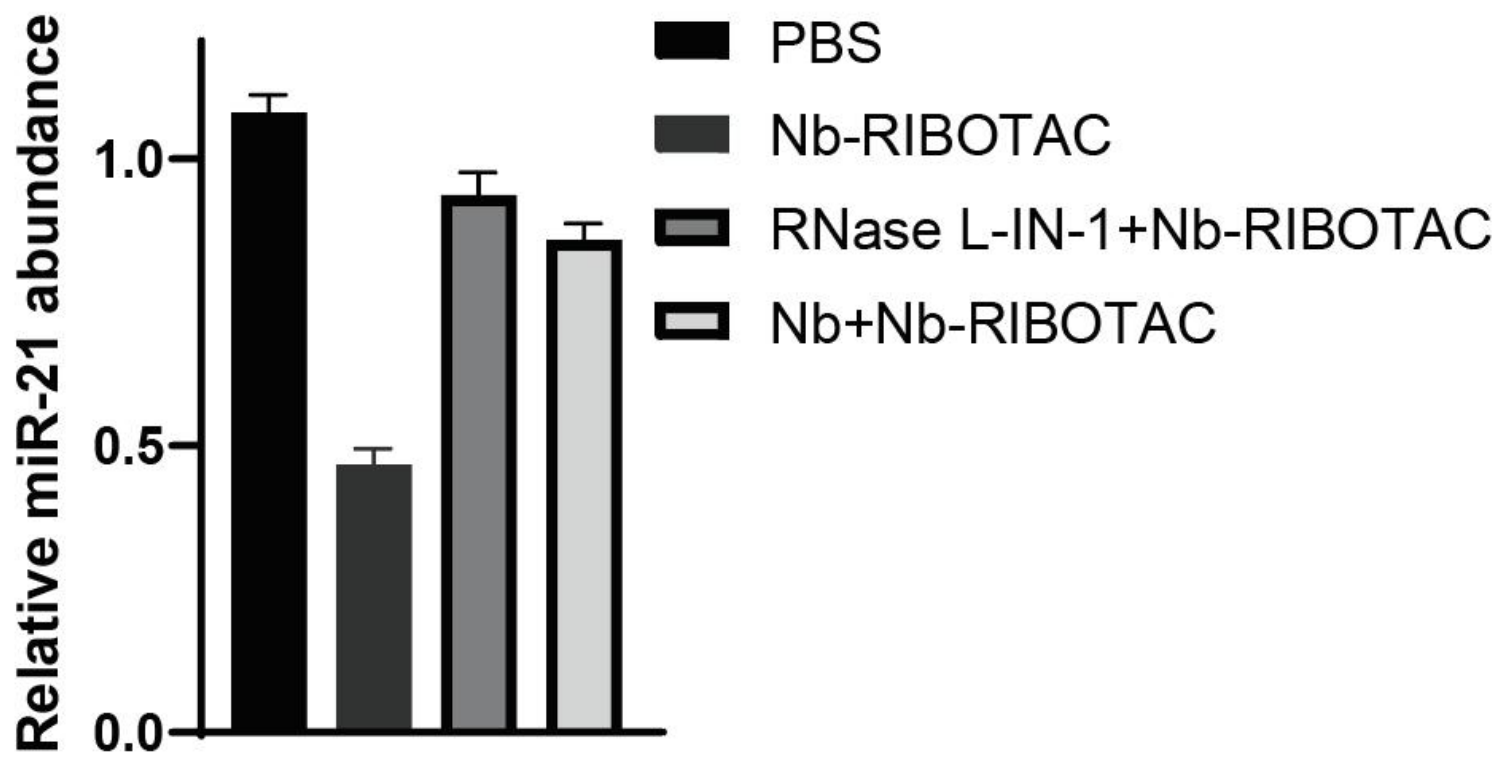

Figure S12. RNase L and EGFR are required for Nb-RIBOTAC-mediated miR-21 knockdown. PANC-1 cells were treated as indicated for 24 h, and miR-21 levels were analyzed by qPCR. miR-21 knockdown by Nb-RIBOTAC (100 nM) was blocked by RNase L inhibitor (10 μM) and by EGFR nanobody competition (1 μM) showed no effect. Data are mean ± SD (n = 3).

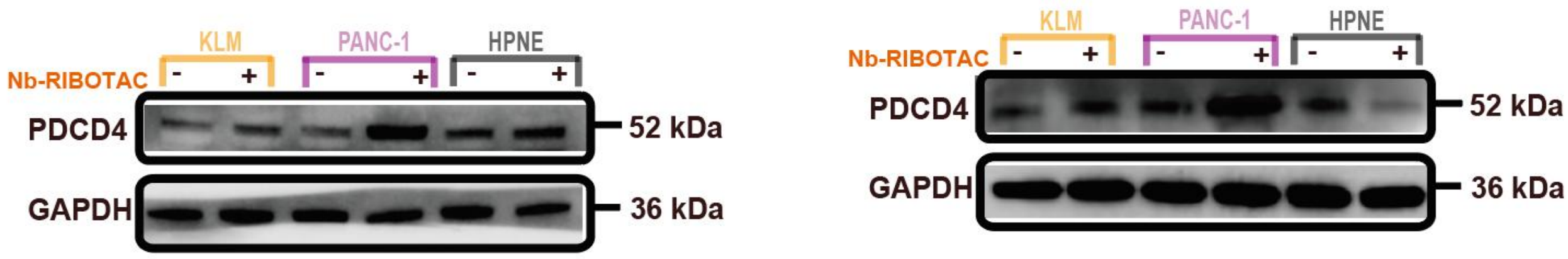

Figure S13. Full replicates of Figure 3g.

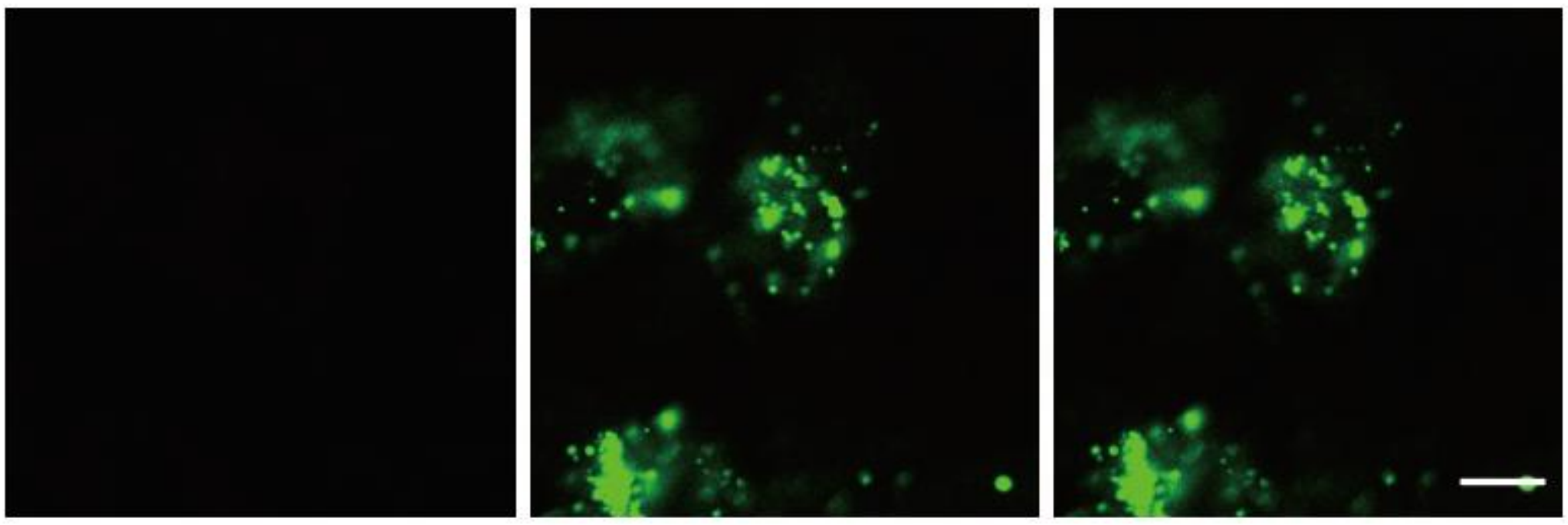

Figure S14. EGFR+ PANC-1 cells were treated with AF647-labeled non-targeting IgG (100 nM) for 1 h. Cells were then labeled with LysoTracker Green (50 nM, 30 min). Shown is a representative frame from live imaging of cells via confocal microscopy. Scale bar = 20 µm.

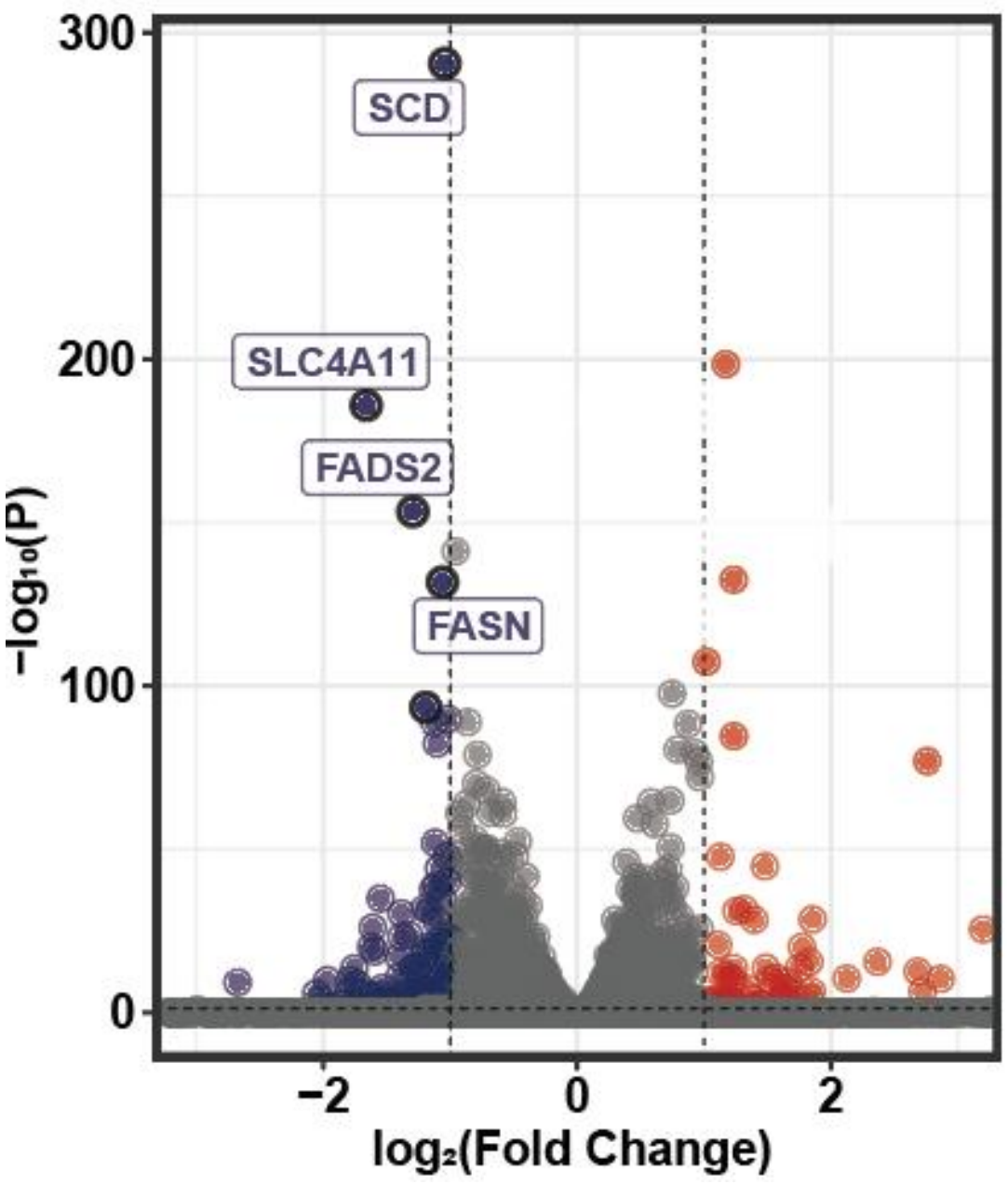

Figure S15. Volcano plot illustrates differential expression between Nb-RIBOTAC-treated and untreated PANC-1 cells.

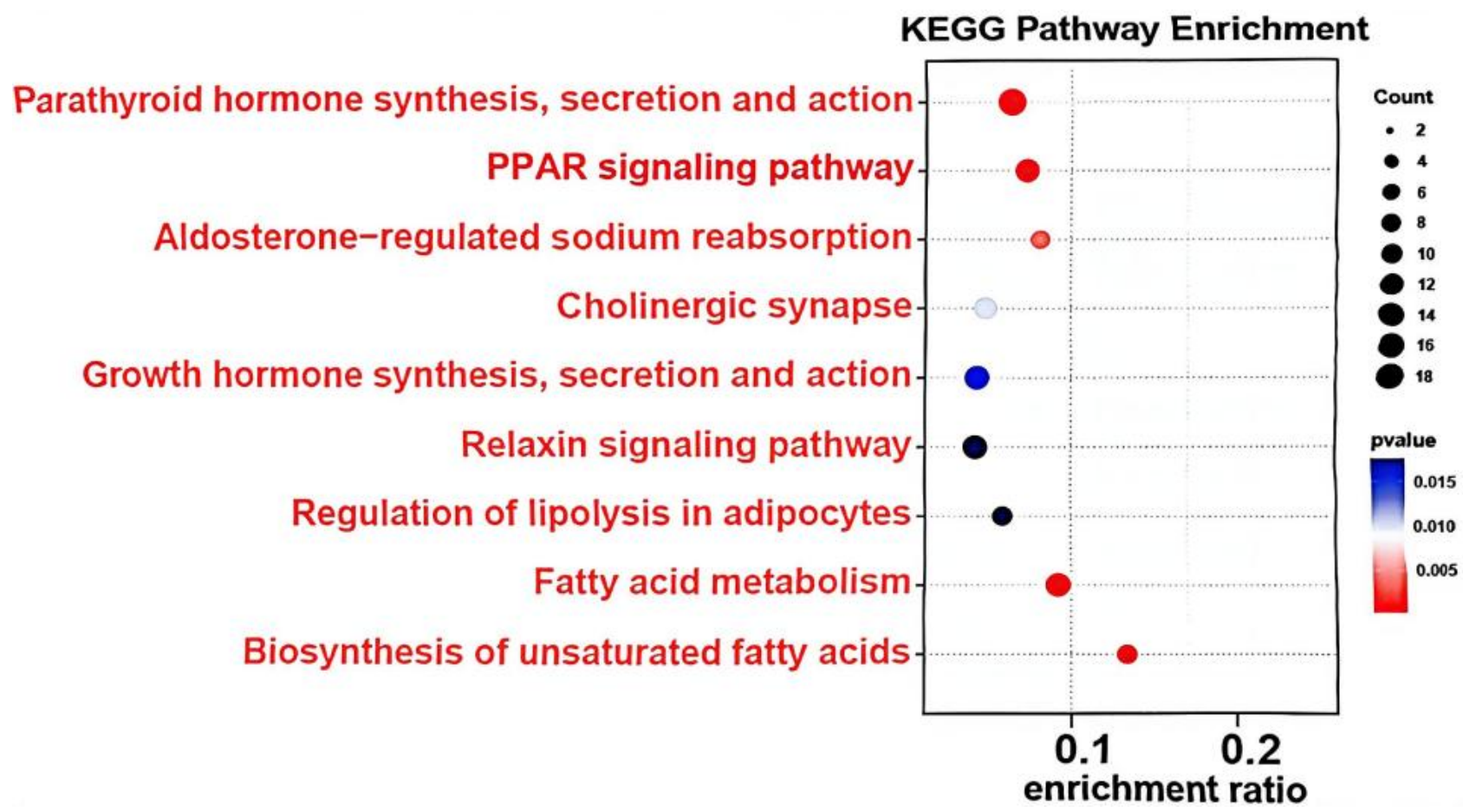

Figure S16. KEGG pathway enrichment analysis. Bar graph showing top nine significantly enriched pathways (FDR<0.01) from RNA-seq data.

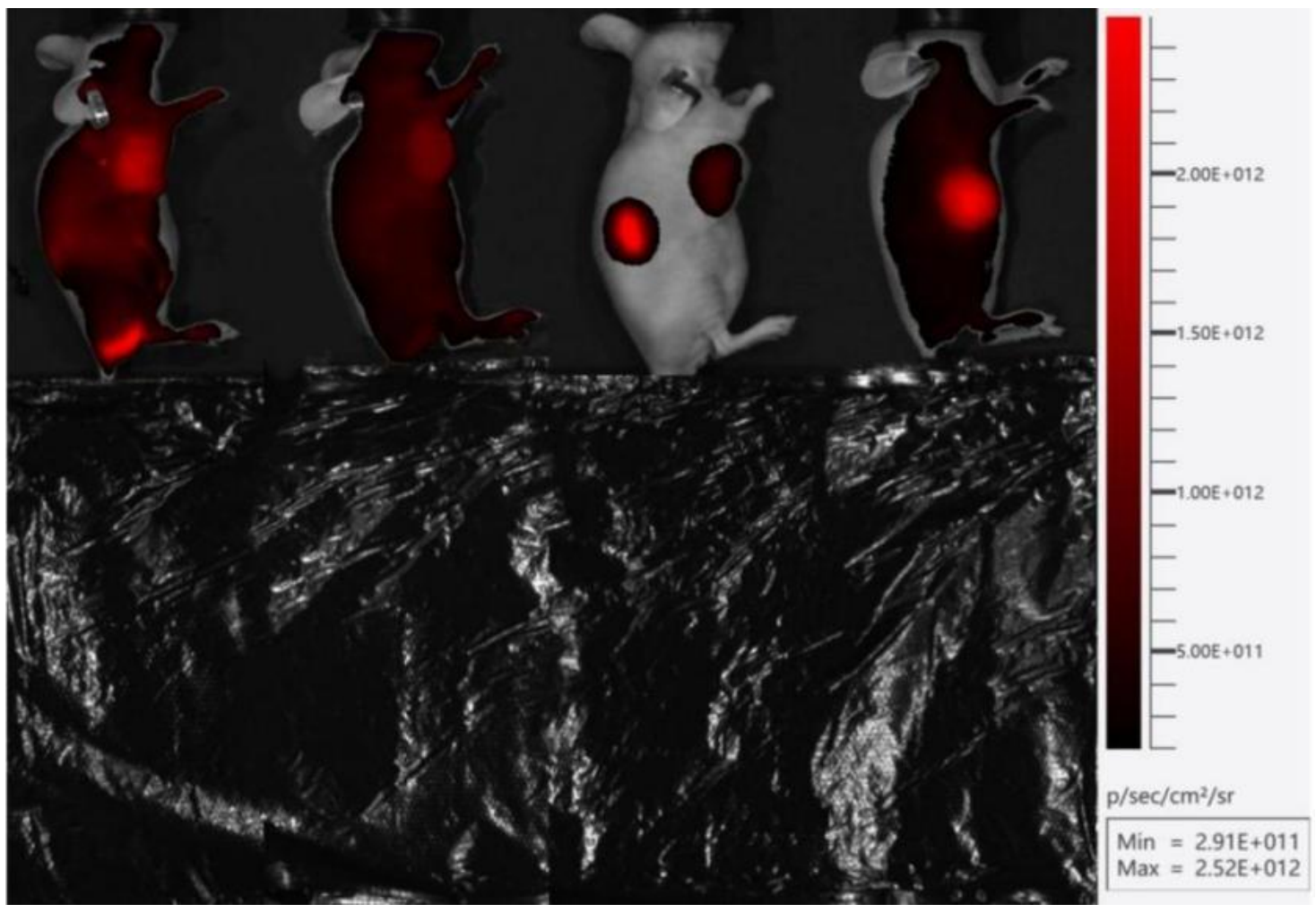

Figure S17. *In vivo* fluorescence imaging of Nb-RIBOTAC in PANC-1 xenograft models at 3, 6, 24, 48h.

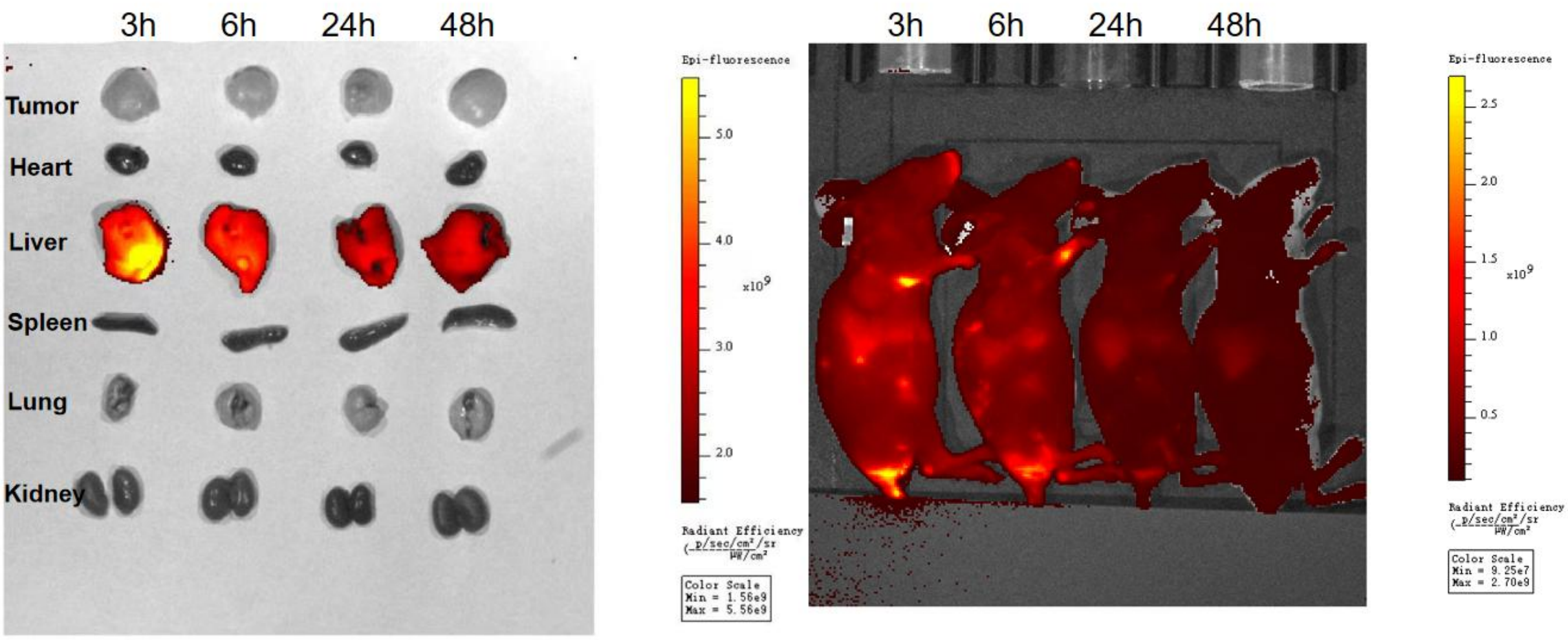

Figure S18. *In vivo* fluorescence imaging of cetuximab in PANC-1 xenograft models at 3, 6, 24, 48h.

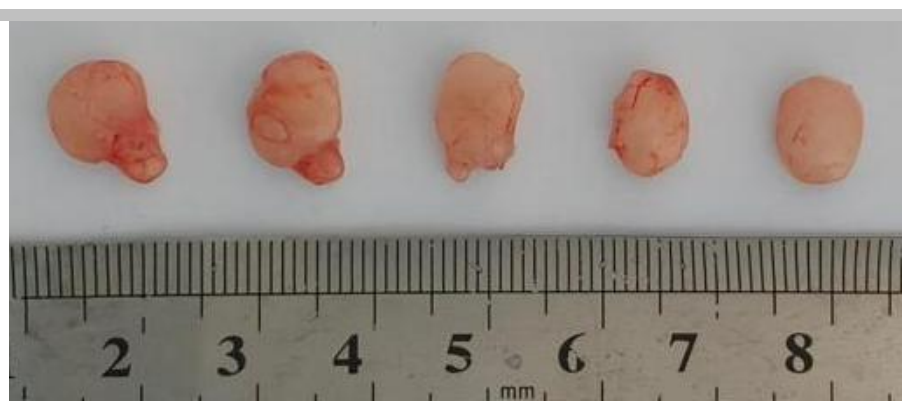

Figure S19. Tumor autopsy results of gemcitabine group of mice at the end of the experiment (n=5 mice/group).

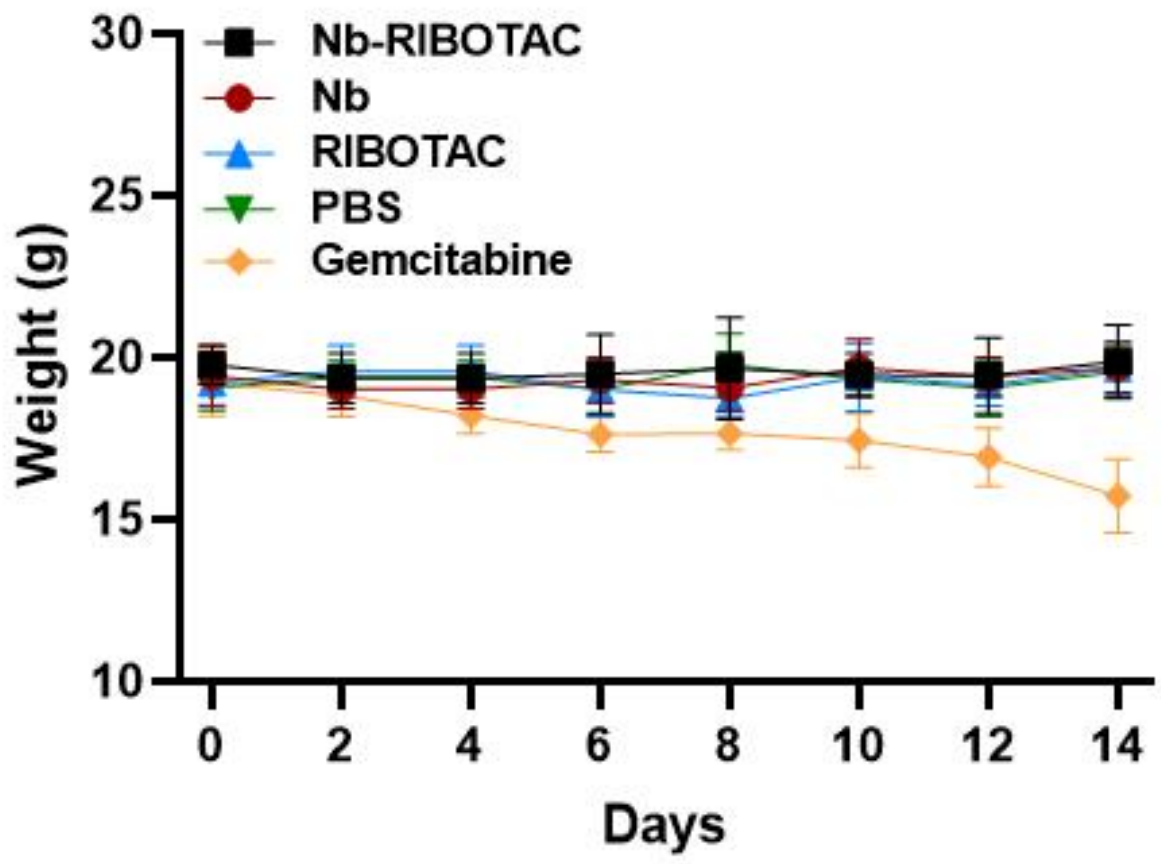

Figure S20. Changes in the body weights of mice over the treatment period. Data represented as mean ± s.d. (n = 5).

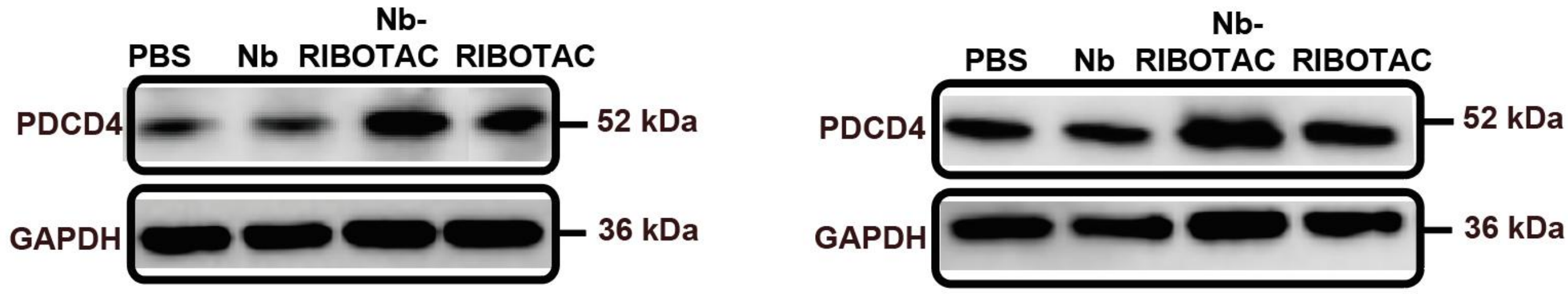

Figure S21. Full replicates of Figure 5f.

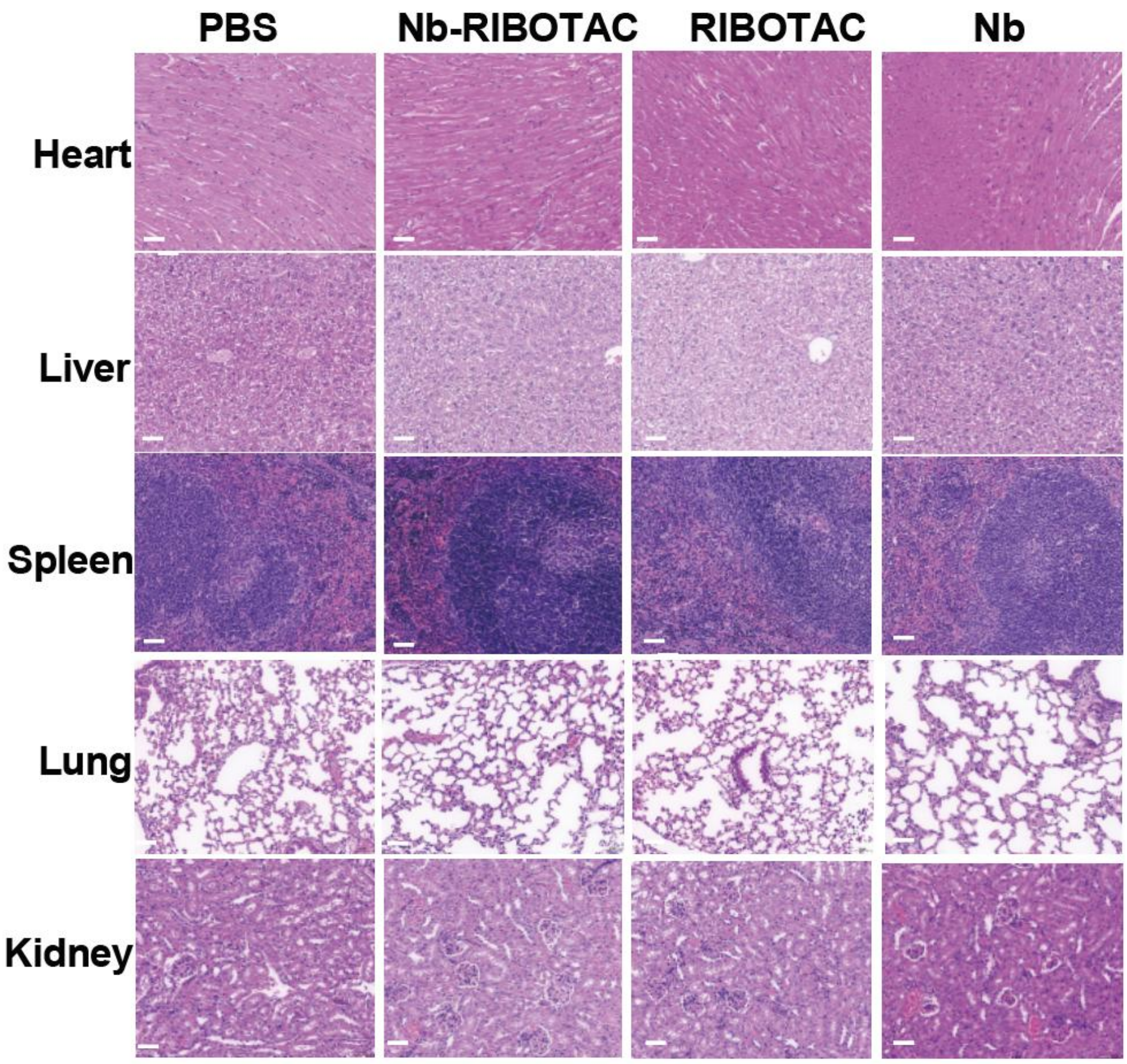

Figure S22. H&E analysis. Major organs were harvested from PANC-1 tumor-bearing mice with different treatments as indicated for H&E staining. Scale bar: 200 µm.

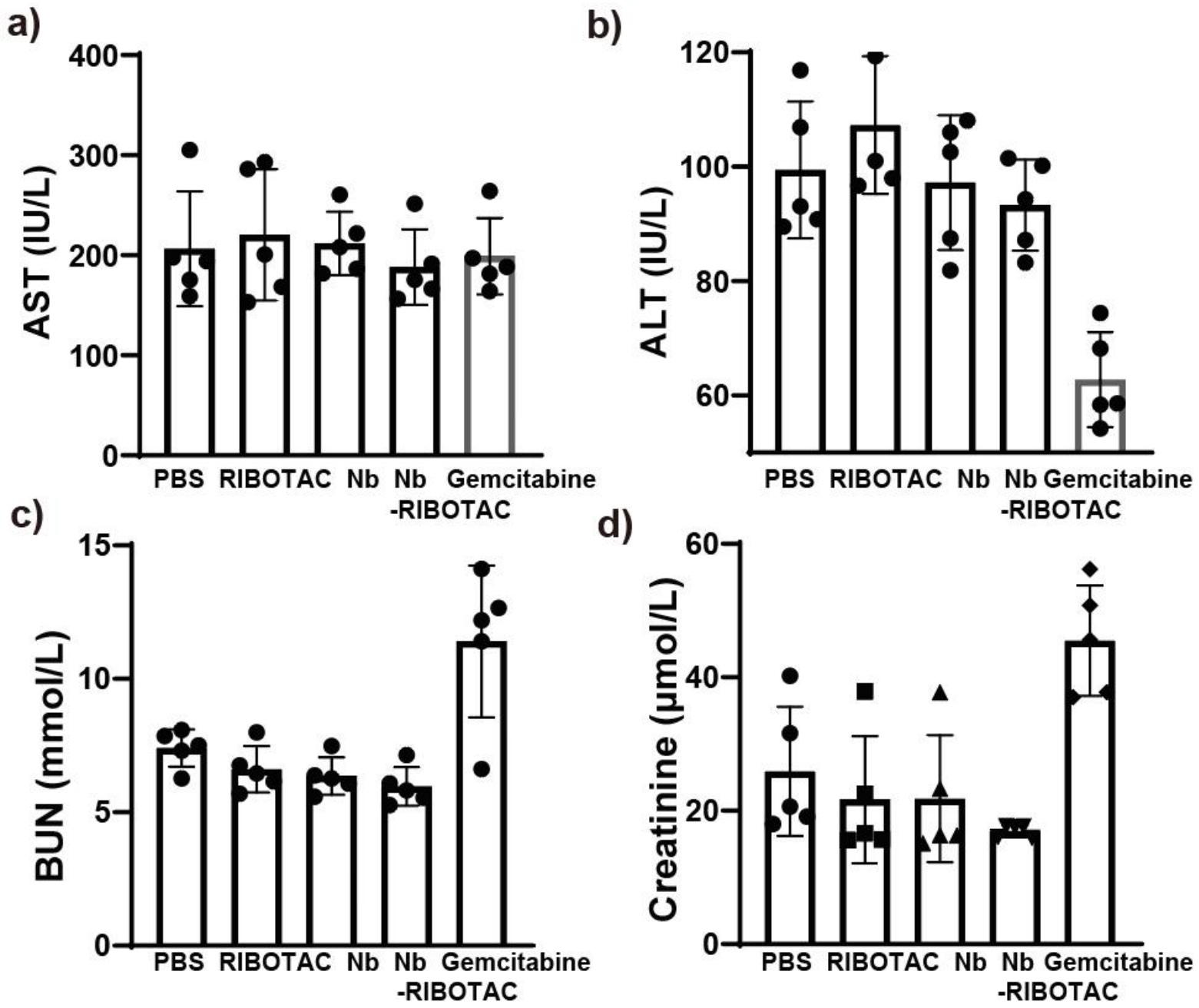

Figure S23. Liver function analysis of PANC-1 tumor-bearing mice received different treatment as indicated. **(a)** Aspartate transaminase (AST), **(b)** alanine aminotransferase (ALT) and **(c)** BUN and **(d)** creatinine assay for studying the kidney damage of mice with different treatments as indicated. Data are presented as mean ± SD (n = 5).

References

1. Kozomara, A., Birgaoanu, M. & Griffiths-Jones, S. miRBase: from microRNA sequences to function. *Nucleic Acids Res.* **47**, D155–D162 (2019).
2. Friedländer, M. R., Mackowiak, S. D., Li, N., Chen, W. & Rajewsky, N. miRDeep2 accurately identifies known and hundreds of novel microRNA genes in seven animal clades. *Nucleic Acids Res.* **40**, 37–52 (2012).
